# Health for sale: the medicinal plant markets in Trujillo and Chiclayo, Northern Peru

**DOI:** 10.1186/1746-4269-3-37

**Published:** 2007-12-10

**Authors:** Rainer W Bussmann, Douglas Sharon, Ina Vandebroek, Ana Jones, Zachary Revene

**Affiliations:** 1 Head and William L. Brown Curator of Economic Botany, Wm. L. Brown Center, Missouri Botanical Garden, PO Box 299, St. Louis, MO 63166-0299, USA; 2 San Diego Museum of Man, 1350 El Prado, San Diego, CA 94804, USA; 3 Institute of Economic Botany, The New York Botanical Garden, Bronx River Parkway at Fordham Road, Bronx, New York, USA; 4 University of California at Berkeley, Berkeley, CA, 94720, USA; 5 12413 Pleasant Run Terrace, Richmond, VA, 23233, USA

## Abstract

Traditional methods of healing have been beneficial in many countries with or without access to conventional allopathic medicine. In the United States, these traditional practices are increasingly being sought after for illnesses that cannot be easily treated by allopathic medicine. More and more people are becoming interested in the knowledge maintained by traditional healers and in the diversity of medicinal plants that flourish in areas like Northern Peru. While scientific studies of medicinal plants are underway, concern has arisen over the preservation of both the large diversity of medicinal plants and the traditional knowledge of healing methods that accompanies them. To promote further conservation work, this study attempted to document the sources of the most popular and rarest medicinal plants sold in the markets of Trujillo (Mayorista and Hermelinda) and Chiclayo (Modelo and Moshoqueque), as well as to create an inventory of the plants sold in these markets, which will serve as a basis for comparison with future inventories. Individual markets and market stalls were subjected to cluster analysis based on the diversity of the medicinal plants they carry. The results show that markets were grouped based on the presence of: (1) common exotic medicinal plants; (2) plants used by laypeople for self-medication related to common ailments ("everyday remedies"); (3) specialized medicinal plants used by *curanderos *or traditional healers; and (4) highly "specialized" plants used for magical purposes. The plant trade in the study areas seems to correspond well with the specific health care demands from clientele in those areas. The specific market patterns of plant diversity observed in the present study represent a foundation for comparative market research in Peru and elsewhere.

## Introduction

Northern Peru is what Peruvian anthropologist Lupe Camino calls the "health axis" of the old Central Andean culture area stretching from Ecuador to Bolivia [1]. The traditional use of medicinal plants in this region, which encompasses in particular the Departments of Piura, Lambayeque, La Libertad, Cajamarca, and San Martin, dates back as far as the first millennium B.C. (north coastal Cupisnique culture) or at least to the Moche period (AC 100–800), with healing scenes and healers frequently depicted in ceramics [2]. Early ethnobotanically oriented studies focused mainly on the famous "magical" and "mind altering" flora of Peru. A first study on "*cimora*" -another vernacular name for the San Pedro cactus (*Echinopsis pachanoi*) dates back to the 1940's [3]. The first detailed study on a hallucinogen in Peru focused also on San Pedro, and tree datura (*Brugmansia *spp.) [4-6]. A variety of works on these species followed [7,8]. Coca (*Erythroxylum coca*) also attracted early scientific attention [9-13], as did the Amazonian Ayahuasca (*Banisteriopsis caapi*) [14-16]. Chiappe et. al [17] were the first to attempt an overview on the use of hallucinogens in shamanistic practices in Peru. General ethnobotany studies in Peru and Bolivia focused mostly on Quechua herbalism of the Cusco area [18-22]. Other comprehensive studies centered on the border region of Peru and Bolivia around Lake Titicaca [23-26] and the Amazon [27-29]. Northern Peru, in contrast, has always been in the shadow of these more touristically important regions, and very few studies have been conducted to date [30-33]. During the last years, the contemporary use of plants by local healers (*curanderos) *in Northern Peru has been well documented [34-38].

However, as in other areas worldwide, both, healers as well as the population in general, buy their medicinal plants often in local markets. In contrast to the wealth of available ethnobotanical information, literature on local markets, market flows and the value of the plant material traded is rather scarce. Although the value of the international market for medicinals receives high interest [39,40], and ecosystem services as well as non-timber forest products do receive some attention [41-45], studies on the trade volumes of local medicinal markets hardly exist, and mostly focus on the export market [46-48].

Latin America is still terra incognita with regards to its medicinal plant markets, and detailed studies are of high importance because even the local health care authorities start propagating complementary alternative medicine [49]. Macía, García & Vidaurre [50] provided the first study of a high Andean market, focusing on La Paz, Bolivia. However, no data on plant origin and sales volumes were given.

Thus, virtually no information exists so far on the composition of the market flora, the origin of the plant material, and the quantities of plant material sold, and the present study attempts to fill this gap by providing an inventory of medicinal plants of the local markets in Northern Peru, and delineating the most important areas for conservation efforts. In addition, we provide a comparative analysis of these markets based on their similarities and dissimilarities in the plant species being sold.

## Methods

### Surveys

Surveys focusing on medicinal plants sold and their properties were conducted at the markets in Trujillo (Mayorista and Hermelinda) and Chiclayo (Modelo and Moshoqueque) each summer (June/August) from 2001–2006. In June–July 2007 an additional questionnaire including questions about plant origin, pricing and quantities sold was included in the follow-up survey. Surveys were conducted in Spanish by fluent speakers. Surveyors would approach the vendors and explain the premise for the study, including the goal of conservation of medicinal plants in the area. All vendors were asked to participate, but due to expected resistance information could not collected from everyone. From those who gave their prior informed consent, information was collected regarding their inventory of medicinal plants. The vendors were also asked to list the ten most commonly sold plants, and ten plants that were disappearing from the market. Of the plants that were most commonly sold and declining, information was also collected on the location (*montaña *– mountain forest, *costa *– coast, *sierra *– highlands, or *selva *– jungle), origin (*pueblo *– village), cost per unit sold (*soles*), units sold per week (*bultos *– bundles, *paquetes *– packets), the time when the vendors' suppliers distribute goods, and any other information concerning the popularity of the plant. At each market, the number of medicinal plant vendors was counted to estimate how the vendors who participated in the study were representative for the entire market.

### Voucher collection

The specimens are registered under the collection series "RBU/PL," "ISA," "GER," "JULS," "EHCHL," "VFCHL," "TRUBH," and "TRUVANERICA," depending on the year of fieldwork and collection location. Vouchers of all specimens were deposited at the Herbario Truxillensis (HUT, Universidad Nacional de Trujillo) and Herbario Antenor Orrego (HAO, Universidad Privada Antenor Orrego Trujillo). In order to recognize Peru's rights under the Convention on Biological Diversity, especially with regard to the conservation of genetic resources in the framework of a study on medicinal plants, the identification of the plant material was conducted entirely in Peru. No plant material was exported in any form whatsoever.

### Nomenclature

The nomenclature of plant families, genera, and species follows the Catalogue of the Flowering Plants and Gymnosperms of Peru [51]. The nomenclature was compared to the TROPICOS database. Species were identified using the available volumes of the Flora of Peru [52], as well as [53-55], and reference material in the herbaria HUT and HAO. Species and author names for all species encountered are given in Additional file 1. For a complete overview on traditional uses, recipes, and botanical vouchers collected see Bussmann and Sharon [34,35].

### Market turnover of plant material

In order to assess the amount of plant material sold, vendors were asked to estimate the daily and weekly amount of material sold for every species in their inventory. For further analysis, vendors were grouped in size-classes according to the number of species in their inventory (1–40, 41–80, >81). Based on this classification, the vendors studied (n = 54) were compared to the total number of vendors in the markets (n = 110), and, based on size class, a total weekly and annual market turnover was calculated for each plant species in the market, and multiplied with the observed sales price for every species, in order to calculate a weekly and annual market value.

### Cluster analysis of markets

The goal of cluster analysis is to group objects together that are similar. Market data were organized in an Excel spreadsheet that contained species as rows and markets as columns. Individual cells contained qualitative presence/absence data, represented by numerical values "1" or "0". The excel spreadsheet was imported into NTSYSpc (version 2.10L) and a (dis)similarity matrix was produced using the Simple Matching coefficient that measures the degree of similarity/dissimilarity between all pairs of markets. Next, a dendrogram (tree) was generated with the UPGMA-SAHN method. Since a cluster analysis will always yield clusters, it is necessary to demonstrate how well the analysis represents the original (dis)similarity matrix. To this end, the tree matrix is transformed into a matrix of ultrametric distances and the latter matrix is statistically compared with the original (dis)similarity matrix. The resulting correlation coefficient "r" between both matrices (normalized Mantel statistic Z) can be used as a measure of goodness of fit for cluster analysis. The degree of fit can be interpreted subjectively as follows: 0.9 = r: very good fit; 0.8 = r < 0.9: good fit; 0.7 = r < 0.8: poor fit; r < 0.7: very poor fit.

## Results and Discussion

**Figure 1:**
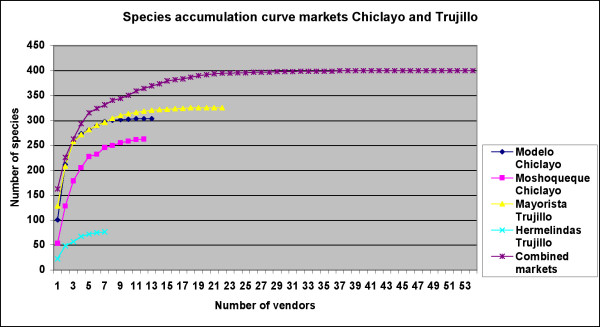
Species accumulation curve for markets studied.

The inventory of 54 of 110 vendors in the Mercados Mayorista and Hermelindas in Trujillo, and Modelo and Moshoqueque in Chiclayo yielded a total of almost 400 medicinal plants and preparations sold at any given day. This was expectedly lower than the reported medicinal flora of the region ([34], 512 species), because many healers interviewed in the former studies rely for their treatments on additional self-collected species or material bought outside the regular market. To evaluate if the current sample size of market vendors represented the complete plant inventory in the various markets, species saturation curves were used (Fig. 1). A 95% species saturation in comparison to the final number of species observed was reached in all markets with the number of vendors studied. This indicates that the sample size was sufficient to capture the market inventory.

### The market inventory

**Figure 2:**
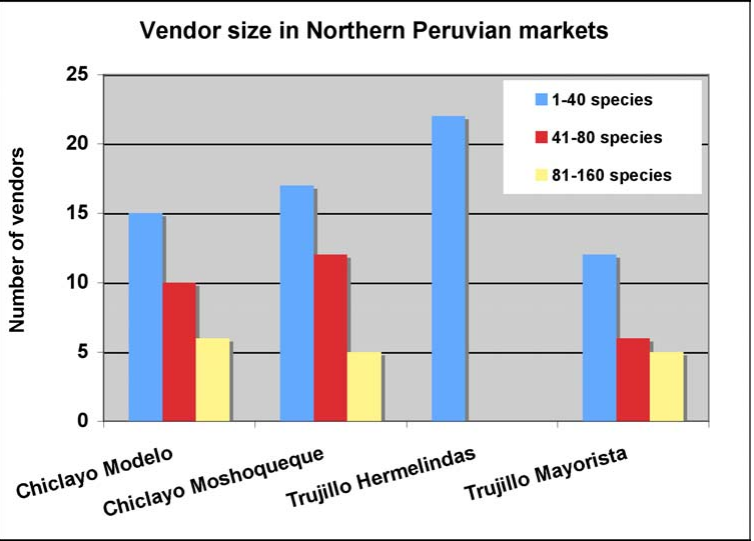
Number and stand size of market vendors.

In the main markets (Modelo, Mayorista and Moshoqueque), the number of small vendors with an inventory of 1–40 plant species, was comparable to the combined number of all larger vendors, and comprised about 50% of the stands. The main exception was Mercado Hermelindas in Trujillo, where only small and very small vendors were present. While the number of mid-size vendors was twice as high as the number of large vendors in the Chiclayo markets, the two groups were about equal in Merdcado Mayorista in Trujillo (Figure 2).

**Figure 3:**
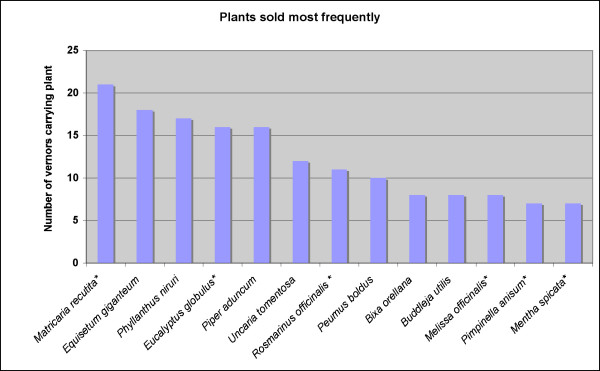
Plants carried most frequently by vendors in Trujillo and Chiclayo (* marks exotic species).

**Figure 4:**
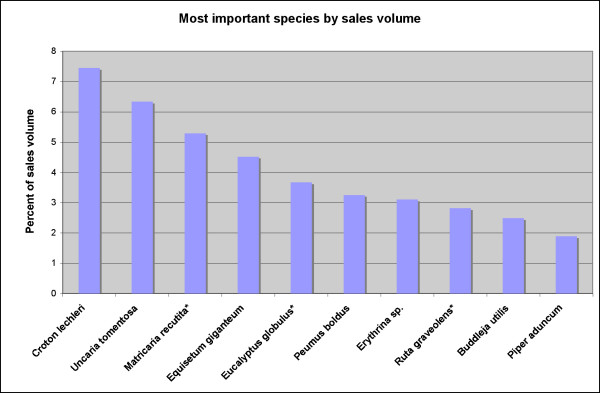
Most important medicinal plant species by sales volume (* marks exotic species).

Exotics played an important role amongst all plants sold. Fifty-nine species (15%) found in all markets were exotics. However, amongst the species most commonly encountered in the inventories, 40–50% were exotics. *Matricaria recutita *(chamomile) was found in the inventory of approximately 70% of vendors. The next most popular species sold in these markets included *Equisetum giganteum*, *Phyllanthus urinaria, Phyllanthus stipulatus*, *Phyllanthus niruri *(chanca piedra – stone breaker), *Eucalyptus globulus (eucalyptus), Piper aduncum, Uncaria tomentosa *(cat's claw), *Rosmarinus officinalis *(rosemary)*, Peumus boldus, Bixa orellana *(achiote) and *Buddleja utilis *(Fig. 3). However, when taking sales volume into account, *Croton lechleri *(dragon's blood), *Uncaria tomentosa*, and *Eucalyptus globulus *were clearly the most important species (Fig. 4).

While it was very easy for all vendors to name their most important and frequently sold species, it proofed impossible to get detailed information about species that vendors observed as "rare" or "disappearing". In most cases, vendors mentioned species as rare because they themselves did not sell them, in many cases these plants were very common outside the market (e.g. *Plantago major *or common plantain), or because demand was so low, that it would not have made sense to carry them in their inventories.

**Figure 5:**
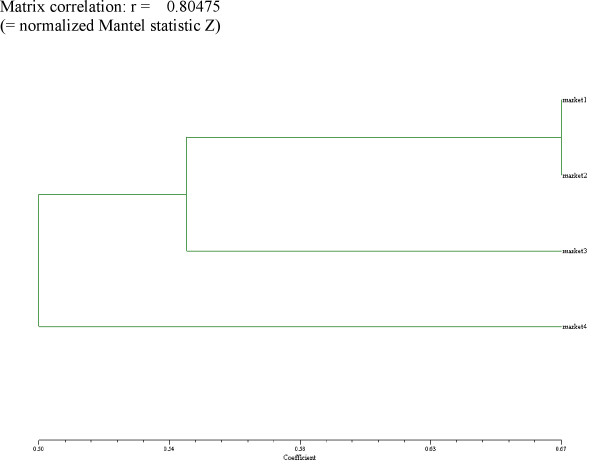
Similarity of individual markets.

A cluster analysis of the four main markets (Fig. 4) indicated that both markets in Chiclayo (market 1 = Modelo, market 2 = Moshoqueque) were expectedly closest related to each other, with Mercado Mayorista in Trujillo (market 3) being next similar, and Mercado Hermelindas (market 4) forming a branch of its own. The dendrogram represents a good fit (Matrix correlation r = 0.805; Normalized Mantel statistic Z). This result is little surprising. Hermelindas contained mostly very small vendors (carrying less than xx plant species). Their inventories represented the most common medicinal plants available, and excluded most species in the large "witchcraft" segment of the pharmacopoeia. On the other hand, the large markets in Trujillo and Chiclayo (Mayorista, Modesto and Mosahoqueque) had several well-established large stands specializing in supplies for healers (including "magical" plants), and thus the inventories of the main markets were relatively similar (Fig. 5). Mercado Mayorista in Trujillo proved to be the most species rich market (325 species and preparations), followed closely by Modelo (305), Moshoqueque (257), and far off Hermelindas (75).

**Figure 6:**
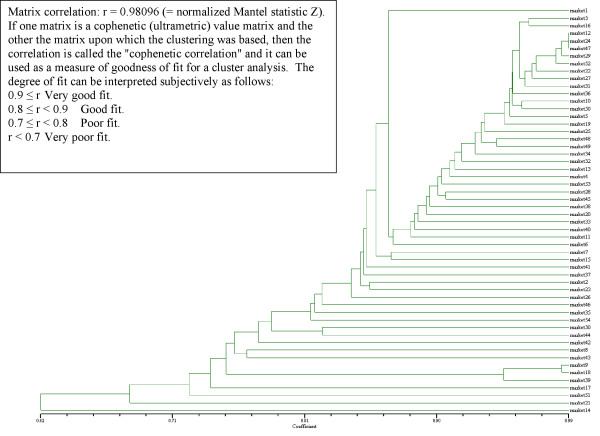
Similarity of vendor portfolios in Trujillo and Chiclayo.

When the 54 vendors in the four main marketplaces are considered individually, 4 large clusters appear which form several subgroups. The dendrogram represents a very good fit (Matrix correlation r = 0.981; Normalized Mantel statistic Z). (Fig. 6):

Cluster 1 (markets 3–19) includes small vendors from Mercado Mayorista in Trujillo (3–31) and vendors from all other markets with a similar portfolio. These stands had a very limited inventory comprising especially the most common exotic species used medicinally.

Cluster 2, including market 1 and 25-6 groups vendors from all 4 markets, but has a high emphasis on all vendors from Mercado Hermelindas in Trujillo. This segment of small vendors carries the 20–30 most important medicinal species and little else, and the portfolios overlap widely. These were, so to say, the vendors for the "everyday cure" where patients could find plants for herbal decoctions they can prepare themselves (e.g. for common cold) without consulting a traditional healer.

In contrast, cluster 3 incorporates mid size vendors who carry the regular pharmacopoeia, plus species needed by traditional healers (*curanderos*) for the treatment of more complicated illnesses. Most vendors in Mercado Moshoqueque fell in this category. However, their respective plant portfolios were often lacking specific "magic" and hallucinogenic species.

Cluster 4 grouped markets that sell species for the real "curing specialists." Markets 30–42 (Modelo), 8–43 (Moshoqueque) and 9–39 (Mayorista) represent clear sub-clusters of vendors in one given market. This indicates that the healers of a given city or suburb use a very well defined pharmacopoeia, which is provided by specialized vendors. The remaining 4 markets (14, 17, 21 and 51) represent the true *curandero *suppliers in the two biggest markets. Markets 51 and 21 (and 14 and 17) were the two biggest stands in Mayorista Trujillo, and Modelo Chiclayo, respectively. All four markets had inventories containing more than 50% of all inventoried plant species, but lacked many of the "generalist" plants sold by other vendors. The portfolio of these stands focused almost entirely on "magical" species that are needed to cure illnesses like "*susto*" (fright), "*mal aire*" (evil wind), "*daño*" (damage), "*envidia*" (envy) and other "magical" or psychosomatic ailments [34]. At the same time, all four vendors catered also to the esoteric tourism crowd that tends to frequent the large markets, and carried a variety of plants that were not used by *curanderos*, but instead were sold to meet tourist demand. Markets 14 and 17 were of particular interest. These represent the stands of two brothers, who, together, carried almost the complete inventory of Mercado Modelo, and as such were the most distinguished specialist vendors of the region.

### A look on sustainability – how much plant and for which price?

An overview of the observed and calculated value of the medicinal plants traded annually in the organized markets of Trujillo and Chiclayo is given in Additional file 2. More than two thirds of all species sold were claimed to originate from the highlands (*sierra*), above the timberline, which represents areas often heavily used for agriculture and livestock grazing.

The overall value of medicinal plants in these markets reaches a staggering 1.2 million US$/year. This figure only represents the share of market vendors, and does not include the amount local healers charge for their cure. Thus, medicinal plants contribute significantly to the local economy. Such an immense market raises questions of the sustainability of this trade, especially because the market analysis does not take into account any informal sales.

Most striking was the fact that 7 indigenous and 3 exotic species, i.e. 2.5% of all species traded, accounted for more than 40% of the total sales volume (with 30 and 12% respectively). Moreover, 31 native species accounted for 50% of all sales, while only 16 introduced plants contributed to more than a quarter of all material sold. This means that little over 11% of all plants in the market accounted for about three fourths of all sales. About one third of this sales volume includes all exotic species traded. None of these are rare or endangered. However, the rising market demand might lead to increased production of these exotics, which in turn could have negative effects on the local flora.

A look at the indigenous species traded highlights important conservation threats. *Croton lechleri *(dragon's blood), and *Uncaria tomentosa *(cat's claw) are immensely popular at a local level and each contributes to about 7% to the overall market value. Both species are also widely traded internationally. The latex of *Croton *is harvested by cutting or debarking the whole tree. *Uncaria *is mostly traded as bark, and again the whole plant is normally debarked. *Croton *is a pioneer species, and apart from *C. lechleri *a few other species of the genus have found their way in the market. Sustainable production of this genus seems possible, but the process has to be closely monitored, and the current practice does not appear sustainable because most *Croton *is wild harvested. The cat's claw trade is so immense, that in fact years ago collectors of this primary forest liana started complaining about a lack of resources [56] and during the years of this study other *Uncaria *species, or even *Acacia *species have appeared in the market as "cat's claw" (own observation). As such, the *Uncaria *trade is clearly not sustainable.

Some of the other "most important" species are either common weeds (e.g. *Desmodium molliculum*), or have large populations (e.g.*Equisetum giganteum*). However, a number of species are very vulnerable. *Tillandsia cacticola *grows in small areas of the coast as epiphyte [51]. The habitat, coastal dry forest and shrub, is heavily impacted by urbanization and mechanized agriculture, the impact of the latter worsened by the current bio-fuel boom. *Gentianella alborosea, G. bicolor, G. graminea, Geranium ayavacense *and *Laccopetalum giganteum *are all high altitude species with very limited distribution. Their large-scale collection is clearly unsustainable, and in the case of *Laccopetalum *collectors indicate that supply is harder and harder to find. The fate of a number of species with similar habitat requirements raises comparable concern. The only species under cultivation at this point are exotics, and a few common indigenous species.

## Conclusion

Northern Peru occupies the middle sector of the "health axis" of the Central Andean cultural area stretching from Bolivia to Ecuador. Few other places on the planet boast a medicinal flora as rich as this region. Archaeological evidence traces the use of plants used in traditional healing and divination practices of the region back at least 2,000 years. Current research indicates that the composition of the local pharmacopoeia has changed since colonial times [38]. However, the overall number of medicinal plants employed seems to have increased. This indicates that the Northern Peruvian health tradition is still going strong, and that the healers are constantly experimenting with new remedies. Recent indications of this are the appearance of Noni (*Morinda citrifolia*) products in large quantities in plant pharmacies and markets in the region. This makes it quite obvious that local herbalists are carefully watching international health trends to include promising species in their own repertoire.

The knowledge of medicinal plants is still taught orally, with no written record. An illustrated identification guide for the medicinal plants of Northern Peru and their uses [35] will hopefully help to keep the extensive traditional knowledge of this area alive. However, Traditional Medicine is experiencing increasing demand, even from a Peruvian perspective as indicated by the fact that the number of herb vendors, in particular in the markets of Trujillo, has increased in recent years, and the markets have an immense economic value. Also a wide variety of medicinal plants from Northern Peru can be found in the global market. While this trend might help to maintain traditional practices and to guarantee traditional knowledge the respect it deserves, it poses a serious threat, as signs of over-harvesting of important species are becoming increasingly apparent.

Today the most serious threat to this millennial tradition is the destruction of medicinal plant habitats. Urban sprawl and the sugar industry have already greatly altered the coastal plains around Trujillo and Chiclayo. Climatic change and deforestation are threatening the mountain forest systems that are the source of many medicinal species. Most importantly, the high Andean ecosystems and sacred lagoons where many medicinally active species are found are in danger of being destroyed by large-scale mining activities [57,58].

## Competing interests

The author(s) declare that they have no competing interests.

## Supplementary Material

Additional File 1 Medicinal plant species sold in the Trujillo and Chiclayo Markets Click here for file

Additional File 2 Quantities of medicinal plants traded in Trujillo and Chiclayo markts (species with more than 1% total sales volume **BOLD**, exotic species RED) Click here for file
