# Supplementary material for: Health for sale: the medicinal plant markets in Trujillo and Chiclayo, Northern Peru
Source: J Ethnobiol Ethnomed. 2007 Dec 10;3:37. doi: 10.1186/1746-4269-3-37 (PMC2245918; doi:10.1186/1746-4269-3-37)
Supplement: Additional File 1 — Medicinal plant species sold in the Trujillo and Chiclayo Markets [file 1746-4269-3-37-S1.pdf]

Additional file 1. Medicinal plant species sold in the Trujillo and Chiclayo Markets

Family	Scientific Name	Common Name
ACANTHACEAE	*Fittonia* spp.	Motalio
ADIANTACEAE	*Adiantum concinnum* Wild. ex H.B.K.	Culantrillo
ADIANTACEAE	*Pellaea ternifolia* C. Chr.	Cuti Cuti
AIZOACEAE	*Tetragonia crystallina* L'Herit	Hierba de la Señorita
ALGAE	*Giartina* spp.	Algae Marinas
ALGAE	*Nostoc* spp.	Algas
ALSTROEMERIACEAE	*Bomarea dulcis* (Hook.) Beauv.	Hierba de Gallo
AMARANTHACEAE	*Alternanthera brasiliana* (L.) Kuntze	Hierba del Oso (Veronica)
AMARANTHACEAE	*Alternanthera halmifolia* (Lam.) Standley & Pittier	Morada
AMARANTHACEAE	*Alternanthera porrigens* (Jacquin) Kuntze	Moradilla
AMARANTHACEAE	*Iresine herbstii* Lindley	Colores (Zangurache)
AMARYLLIDACEAE	*Eustephia coccinea* Cav.	Para Para (Puma Para)
AMARYLLIDACEAE	*Stenossemon flammidum* Raf.	Cholo Lindo
ANACARDIACEAE	*Mauria heterophylla* H.B.K.	Chacur (Feregreco, Hierba Sanchez, Tres Hojas, Trinidad)
ANACARDIACEAE	*Schinus molle* L.	Molle
ANNONACEAE	*Annona muricata* L.	Hoja de Huanabana
APIACEAE	*Ammi visnaga* (L.) Lam.	Bisnaga
APIACEAE	*Apium graveolens* L.	Apio
APIACEAE	*Arracacia xanthorrhiza* Bancroft	Racacha
APIACEAE	*Coriandrum sativum* L.	Culantro
APIACEAE	*Daucus montanus* H. & B. ex Spreng.	Hierba de Zorrillo (Zanaoria de Zorro)
APIACEAE	*Foeniculum vulgare* P. Miller	Hinojo (Hojas de Anis)
APIACEAE	*Petroselinum crispum* (Miller) A.W. Hill	Perejil
APIACEAE	*Pimpinella anisum* L.	Anis (Anis Criollo)
APOCYNACEAE	Mandevilla cf. *trianae* Woodson	Bejuco
APOCYNACEAE	*Nerium oleander* L.	Laurel
APOCYNACEAE	*Plumeria rubra* L.	Pachuli
APOCYNACEAE	*Thevetia peruviana* (Pers.) Schum.	Cabalonga (Camalonga, Maichil)
APOCYNACEAE	*Vallesia glabra* (Cav.) Link.	Cuncuno
AQUIFOLIACEAE	*Ilex guayusa* Loes	Guayusa (Citrodora)
ARALIACEAE	*Oreopanax eriocephalus* Harms	Mano de León, Maque Maque, Mano de Onza
ARALIACEAE	*Panax ginseng* Mey.	Ginseng
ARECACEAE	*Bactris* spp.	Chonta
ASCLEPIADACEAE	*Sarcostemma clausum* (Jacquin) Schultes	Hierba Judia (Marrajudio)
ASPHODELACEAE	*Aloe vera* (L.) Burm f.	Sabila
ASTERACEAE	*Acanthoxanthium spinosum* (L.) Furreau	Juan Alonso, Garcia Alonzo
ASTERACEAE	*Achillea millefolium* L.	Milenrama
ASTERACEAE	*Ambrosia peruviana* Willd.	Ajenjo (Altamis, Llatama Negra Malera, Manzanilla de Muerto, Marco)
ASTERACEAE	*Baccharis genistelloides* (Lam.) Pers.	Karqueja (Simba Simba)
ASTERACEAE	*Baccharis vaccinioides* H.B.K.	Hierba de Sigueme Sigueme
ASTERACEAE	*Bidens pilosa* L.	Amor Seco (Cadillo)
ASTERACEAE	*Calendula officinalis* L.	Calendula
ASTERACEAE	*Chuquiraga spinosa* sp. *huamanpinta* C. Ezcurra	Chuquiragua (Huamanpinta)
ASTERACEAE	*Chuquiragua weberbaueri* Tovar	Amaro
ASTERACEAE	*Cronquistianthus lavandulifolius* DC.	Pulmonaria
ASTERACEAE	*Cynara cardunculus* L.	Alcachofa

**ASTERACEAE**	*Diplostephium sagasteguii* Cuatrecasas	Hierba del Tigre
**ASTERACEAE**	*Eupatorium gayanum* Wedd.	Asma chilca (Chilco)
**ASTERACEAE**	*Flaveria bidentis* (L.) Kuntze	Mata Gusano
**ASTERACEAE**	*Gnaphalium americanum* Mill.	Lechugilla,Queta Queta, Queto Queto, Lengua del Perro
**ASTERACEAE**	*Loricaria ferruginea* (R. & P.) Wedd.	Palmita
**ASTERACEAE**	*Loricaria pauciflora* Cuatr.	Palma
**ASTERACEAE**	*Loricaria thyrsoidea* (Cuatr.) Dillon	Trensilla
**ASTERACEAE**	*Matricaria frigidum* (H.B.K.) Kunth	Manzanilla de olor (Manzanilla Dulce y Amarga)
**ASTERACEAE**	*Matricaria recutita* L.	Manzanilla (Dulce y Amarga), Labanda, Manzanillón
**ASTERACEAE**	*Monactis flaverioides* H.B.K.	Churguis,Hierba del Susto (y Amarilla)
**ASTERACEAE**	*Oritrophium peruvianum* (Lam.) Cuatrec.	China Linda (Hierba del Sol, Vira Vira, Wiña Wiña)
**ASTERACEAE**	*Paranephelius uniflorus* Poepp. & Endl.	Carapa de Chancho (Pacha Rosa)
**ASTERACEAE**	*Perezia multiflora* (H. & B.) Lessing	Escorcionera
**ASTERACEAE**	*Perezia pungens* (H.B.K.) Cass.	Lengua Vaca
**ASTERACEAE**	*Picrosia longifolia* D. Don	Achicoria (Chicoria)
**ASTERACEAE**	*Porophyllum ruderale* (Jacq.) Cass.	Gallinazo (Pata de Gallina)
**ASTERACEAE**	*Pseudogynoxis cordifolia* (Cass.) Cabr.	San Juan
**ASTERACEAE**	*Schkuhria pinnata* (Lam.) Kuntze	Canchalagua (Canchalagua Chica)
**ASTERACEAE**	*Senecio canescens* (H.B.K.) Cuatrecasas	Oreja de Conejo
**ASTERACEAE**	*Senecio chinogeton* Wedd.	Hornamo (Leon) Amarillo
**ASTERACEAE**	*Senecio pseudotites* Grieseb.	Arnica
**ASTERACEAE**	*Smallanthus sonchifolius* (Poepp. & Endl) H. Rob.	Llacon (Yacon)
**ASTERACEAE**	*Sonchus oleraceus* L.	Karqueja, Serraja
**ASTERACEAE**	*Spilanthes leiocarpa* DC.	Turre
**ASTERACEAE**	*Stevia rebaudiana* Bertoni	Estevia
**ASTERACEAE**	*Tagetes elliptica* Sm.	Supequewa, Supiquegua
**ASTERACEAE**	*Tagetes erecta* L.	Clavo de Chino (Claveles Chino, Flores del Muerto, Marigold)
**ASTERACEAE**	*Tagetes filifolia* Lag.	Anis Serrano
**ASTERACEAE**	*Tanacaetum parthenium* (L.) Sch. Bip.	Santa Marta
**ASTERACEAE**	*Taraxacum officinale* Wiggers	Diente de Leon
**ASTERACEAE**	*Tesaria integrifolia* R. & P.	Pajaro Bobo
**ASTERACEAE**	*Trixis cacalioides* H.B.K.	Añasquero Chico
**ASTERACEAE**	*Weddelia latifolia* DC.	Chulgan
**ASTERACEAE**	*Werneria pygmaea* H. & A.	Halago
**BALANOPHORACEAE**	*Heliosis cayennensis* (Swartz) Sprengel	Huanarpo
**BERBERIDACEAE**	*Berberis buceronis* J.F. Macbride	Palo Amarillo
**BETULACEAE**	*Alnus acuminata* H.B.K.	Aliso
**BIGNONIACEAE**	*Crescentia cujete* L.	Tutuma
**BIGNONIACEAE**	*Jacaranda acutifolia* H. & B.	Yarabisca
**BIGNONIACEAE**	*Tabebuia* spp.	Huayacán
**BIGNONIACEAE**	*Tynnanthus scabra* (Hoffm. ex Roem. & Schult.) Schum.	Clavo Huasca
**BIXACEAE**	*Bixa orellana* L.	Achote
**BORAGINACEAE**	*Borrago officinalis* L.	Borraja
**BORAGINACEAE**	*Cordia alliodora* (R. & P.) Oken	Ajo Quiro, Ajo Sacha, Ajojero
**BORAGINACEAE**	*Cordia lutea* Lam.	Flor de Overo
**BORAGINACEAE**	*Heliotropium curassavicum* L.	Alacran
**BORAGINACEAE**	*Symphytum* spp.	Confrey
**BORAGINACEAE**	*Tiquilia paronychoides* (Phil.) Rich.	Flor de Arena (Manita de Raton)
**BRASSICACEAE**	*Capsella bursa-pastoris* (L.) Medic.	Bolsa de Pastor
**BRASSICACEAE**	*Cheiranthus cheiri* L.	Alalali

**BRASSICACEAE**	*Lepidium meyenii* Walpers	Maca
**BRASSICACEAE**	*Rorippa nasturtium-aquaticum* (L.) Hayek	Berros (Berruco)
**BROMELIACEAE**	*Puya hamata* L.B. Sm.	Hierba de Carnero, Lana de Carnejo
**BROMELIACEAE**	*Tillandsia cacticola* L.B. Sm.	Palmera (Siempre Viva)
**BURSERACEAE**	*Bursera graveolens* (H.B.K.) Triana & Planchon	Palo Santo
**CACTACEAE**	*Echinopsis pachanoi* (Britton & Rose) Friedrich & G. Rowley	San Pedro
**CACTACEAE**	*Opuntia ficus-indica* (L.) Miller	Tuna, Nopal
**CAMPANULACEAE**	*Siphocampylus angustiflorus* Schlechtendal	Contoya
**CAPPARIDACEAE**	*Capparis crotonoides* H.B.K.	Bichayo
**CAPRIFOLIAEAE**	*Lonicera japonica* Thunberg	Madre Selva
**CAPRIFOLIAEAE**	*Sambucus peruviana* H.B.K.	Sauco (Tilo)
**CARICACEAE**	*Jacartia digitata* (Poepp. & Endl.) Solms-Lang.	Contra Hechizo
**CARYOPHYLLACEAE**	*Dianthus caryophyllus* L.	Clavel (Serrano)
**CHENOPODIACEAE**	*Chenopodium ambrosioides* L.	Paico (Espesote)
**CHLORANTHACEAE**	*Hedyosmum racemosum* (R. & P.) G. Don.	Asarcito
**CHRYSOBALANACEAE**	*Coupeia* sp.	Acharachango
**CLETHRACEAE**	*Clethra castaneifolia* Meissner	Hierba del Olvido
**CLUSIACEAE**	*Hypericum laricifolium* Jus.	Hierba de la Fortuna
**CLUSIACEAE**	*Hypericum silenioides* Jus.	Sentaura
**COMMELINACEAE**	*Tripogandra multiflora* (Sw.) Raf.	Cachurros
**CRASSULACEAE**	*Echeveria peruviana* Meyen	Pim Pim
**CRASSULACEAE**	*Echeveria peruviana* Meyen	Pinpin
**CUCURBITACEAE**	*Momordica balsamina* L.	Balsamina
**CUCURBITACEAE**	*Sicana odorifera* (Vell.) Naud.	Secana
**CUPRESSACEAE**	*Cupresus lusitanica* Miller	Cipres
**CYPERACEAE**	*Cyperus articulatus* L.	Varita de San Jose
**CYPERACEAE**	*Oreobolos goeppingeri* Sues	Carpintero (Hierba del)
**DIOSCOREACEAE**	*Dioscorea tambillensis* Kunth	Papa Semitona
**DIOSCOREACEAE**	*Dioscorea trifida* L.f.	Papa Madre (Papa Pacta)
**DIPSACACEAE**	*Scabiosa atropurpurea* L.	Ambarina (Ambarina Negra)
**EPHEDRACEAE**	*Ephedra americana* H. & B.	Diego Lopez (Suelda con Suelda)
**EQUISETACEAE**	*Equisetum giganteum* (Wedd.) Ulbrich	Cola de Caballo
**ERICACEAE**	*Bejaria aestuans* L.	Buen Querer (Cadillo, Hierba de la Postema, Purenrosa)
**ERICACEAE**	*Gaultheria erecta* Vent.	Mullaca(grande y chico)
**ERICACEAE**	*Gaultheria reticulata* H.B.K.	Maique (Toromaique)
**ERYTHROXYLACEAE**	*Erythroxylon coca* Lam.	Coca
**EUPHORBIACEAE**	*Alchornea castanaeifolia* (Willd.) Jussieu	Ipurura
**EUPHORBIACEAE**	*Croton draconoides* Muell. Arg. *& Croton lechleri* Muell. Arg.	Sangre de Grado
**EUPHORBIACEAE**	*Hura crepitans* L.	Abia, Coco de Abilla
**EUPHORBIACEAE**	*Jatropha curcas* L.	Piñones
**EUPHORBIACEAE**	*Jatropha gosypiifolia* L.	Piñones
**EUPHORBIACEAE**	*Jatropha multifida* L.	Piñones
**EUPHORBIACEAE**	*Manhiot esculenta* Crantz	Yuca
**EUPHORBIACEAE**	*Phyllanthus urinaria* L.	Chanca Piedra
**EUPHORBIACEAE**	*Phyllanthus niruri* L.	Chanca Piedra
**EUPHORBIACEAE**	*Phyllanthus stipulatus* (Raf.) Webster	Chanca Piedra
**EUPHORBIACEAE**	*Ricinus communis* L.	Piñon
**FABACEAE**	*Acacia senegal* (L.) Willd.	Goma Arabia
**FABACEAE**	*Caesalpinia paipai* R. & P.	Pai Pai
**FABACEAE**	*Caesalpinia spinosa* (Molina) Kuntze	Talla (Tara)
**FABACEAE**	*Cajanus cajan* (L.) Millsp.	Chivato
**FABACEAE**	*Cassia fistula* L.	Caña Fistula

**FABACEAE**	*Copaifera paupera* (Herz.) Dwyer.	Aciete de Copaiba
**FABACEAE**	*Desmodium molliculum* (H.B.K.) DC.	Manayupa (Pie de Perro, Pata de Perro, Chancas de Comida)
**FABACEAE**	*Desmodium triflorum* (L.) DC	Pega Pega
**FABACEAE**	*Ormosia* sp.	Huayruro (Semilla)
**FABACEAE**	*Erythrina* spp.	Huailulo, Mishquina
**FABACEAE**	*Lathyrus odoratus* L.	Tacon
**FABACEAE**	*Leucaena leucocephala* (Lam.) De Wit	Arabisca
**FABACEAE**	*Lupinus mutabilis* Sweet	Chocho
**FABACEAE**	*Mimosa albida* H. & B.	Tapa Tapa
**FABACEAE**	*Mimosa polydactyla* H. & B.	Sensitiva
**FABACEAE**	*Mucuna rostrata* Benth.	Habilla (chica y grande)
**FABACEAE**	*Myroxylon balsamum* (L.) Harms.	Palo Balsamo (Quina Quina, Balsamo)
**FABACEAE**	*Senna monilifera* H.S. Irwin & Bowley	Hoja de Sen
**FABACEAE**	*Senna occidentalis* (L.) Link.	Retana (Retania)
**FABACEAE**	*Spartium junceum* L.	Retama
**FABACEAE**	*Trifolium repens* L.	Trebol (Trebol de Agua)
**FABACEAE**	*Zornia reticulata* Sm.	Hierba de la Vibora
**GENTIANACEAE**	*Gentianella alborosea* (Grimes) Pringle	Hercampuri
**GENTIANACEAE**	*Gentianella bicolor* (Wedd.) J. Pringle	Corpus Way (Hornamo Leon)
**GENTIANACEAE**	*Gentianella* crasicaulis J. Pringle	Hojas de Violeta
**GENTIANACEAE**	*Gentianella dianthoides* (H.B.K.) Fabris	Amargon (Genciana)
**GENTIANACEAE**	*Gentianella graminea* (H.B.K.) Fabris	Chinchimali (Sumaran)
**GENTIANACEAE**	*Gentianella* sp.	Anga Macha
**GERANIACEAE**	*Erodium cicutarium* (L.) L'Herit.	Agujilla
**GERANIACEAE**	*Geranium ayavacense* Willd ex H.B.K.	Pachachanga (Pachuchuaca, Miscamisa)
**GERANIACEAE**	*Geranium sesiliflorum* Cavanilles	Pasuchaca (Miscamisca)
**GERANIACEAE**	*Pelargonium odoratisimum* (L.) L'Herit.	Malva de olor (Malva de Oro, Olorosa)
**HIPPOCRATEACEAE**	*Tontelea crassifolia* (Mart.) Spreng.	Bejuco de Montaña
**ILLICIACEAE**	*Illicium verum* Hook. f.	Anis Estrella
**JUGLANDACEAE**	*Juglans neotropica* Diels	Nogal
**LAMIACEAE**	*Hyptis sidifolia* (L´Her.) Briq.	Albaca de Campo (Serrana), Pedorera
**LAMIACEAE**	*Lavandula angustifolia* Miller	Alhucema
**LAMIACEAE**	*Lepechinia meyenii* (Walpers) Epling	Salvia (Real), Salvia Paraguay
**LAMIACEAE**	*Marrubium vulgare* L.	Chancaz de Muerto, Cordon de Muerto, Corontilla
**LAMIACEAE**	*Melisa officinalis* L.	Melisa (Toronjil)
**LAMIACEAE**	*Mentha spicata* L.	Hierba Buena (Menta)
**LAMIACEAE**	*Mentha x piperita* L.	Poleo
**LAMIACEAE**	*Minthostachys mollis* Griesebach	Muña
**LAMIACEAE**	*Ocimum basilicum* L.	Albaca (Albahaca)
**LAMIACEAE**	*Origanum majorana* L.	Mejorana
**LAMIACEAE**	*Origanum vulgare* L.	Oregano
**LAMIACEAE**	*Otholobium glandulosum* (L.) Grimes	Culein
**LAMIACEAE**	*Rosmarinus officinalis* L.	Romero (Romero Castilla)
**LAMIACEAE**	*Salvia discolor* H.B.K.	Llatama
**LAMIACEAE**	*Salvia rosmarinifolia* Hort. ex G. Don.	Romero Blanco (Romero Silvestre y Romero del Campo)
**LAMIACEAE**	*Salvia tubiflora* R. & P.	Chochocon, Hierba del Mal Aire, Paja del Aire
**LAMIACEAE**	*Satureja elliptica* (R. & P.) Briq.	Chipita
**LAMIACEAE**	*Satureja pulchella* (H.B.K.) Briquet	Panisara (Panizara)
**LAMIACEAE**	*Stachys arvensis* L.	Hierba Terrestre
**LAMIACEAE**	*Thymus vulgaris* L.	Tomillo
**LAURACEAE**	*Aniba roseadora* Ducke	Palo Rosa

**LAURACEAE**	*Nectandra floribunda* Nees	Espingo, Ishpingo
**LAURACEAE**	*Nectandra reticulata* (R. & P.) Mez.	Espingo (semilla, flor, amarillo/blanco)
**LAURACEAE**	*Persea americana* Mill.	Palta
**LAURACEAE**		Asmala
**LICHENES**	*Siphula* sp.	Papelillo
**LILIACEAE**	*Allium sativum* L.	Ajo Macho
**LILIACEAE**	*Hesperoziphium niveum* (Rav.) Rav.	Hierba de la Justicia
**LINACEAE**	*Linum sativum* L.	Linaza
**LINACEAE**	*Linum usitatissimum* L.	Linaza
**LOASACEAE**	*Mentzelia cordifolia* Dombey	Anguarate
**LOGANIACEAE**	*Buddleja utilis* Kraenzl.	Flor Blanca
**LYCOPODIACEAE**	*Huperzia crassa* (H. & B. ex Willd.) Rothm.	Condor (Condores)
**LYCOPODIACEAE**	*Lycopodium jusiaei* Desv. ex Poir	Hierba del Hombre
**LYCOPODIACEAE**	*Lycopodium thyoides* H. & B. ex Willd.	Trencilla (Roja)
**LYTHRACEAE**	*Cuphea racemosa* (L.f.) Spreng.	Hierba del Coche
**LYTHRACEAE**	*Cuphea strigulosa* H.B.K.	Hierba de Toro (Lancetilla, Sanguinaria)
**MALPIGHIACEAE**	*Banisteriopsis caapii* (Spruce ex Grieseb.) Morton	Ayahuasca
**MALVACEAE**	*Abelmoschus moschatus* Medikus	Hierba de Culebra
**MALVACEAE**	*Alcea rosea* (L.) Cavanilles	Malva de Lavado
**MALVACEAE**	*Malva parviflora* L.	Malva real (Malva Blanca)
**MELASTOMATACEAE**	*Brachyotum tyrianthium* Macbride	Sarcilleja
**MELIACEAE**	*Trichilia* spp.	Pucho
**MENISPERMACEAE**	*Abuta grandiflora* (Mart.) Sand.	Abuta
**MONIMIACEAE**	*Peumus boldus* Molina	Boldo
**MONIMIACEAE**	*Siparuna muricata* (R. & P.) A. DC.	Añascero (Añasquero, y Añascero chico)
**MORACEAE**	*Brosmium rubescens* Taubert	Murure, Palo Sangre
**MORACEAE**	*Ficus carica* L.	Higo
**MORACEAE**	*Morus alba* L.	Morera
**MUSACEAE**	*Musa x paradisiaca* L.	Beldaco, Platano
**MYRICACEAE**	*Myricaria dubia* (H.B.K.) McVaugh.	Camu Camu
**MYRISTICACEAE**	*Myristica fragrans* L.	Nuez moscada
**MYRTACEAE**	*Eucalyptus globulus* Labill.	Eucalipto
**MYRTACEAE**	*Eugenia obtusifolia* Cambes.	Lanchi (Rumilanchi, Unquia)
**MYRTACEAE**	*Myrcianthes discolor* (H.B.K.) Vaughn, *Myrcianthes fragrans* (Sw) McVaugh	Lanche
**MYRTACEAE**	*Psidium guajava* L.	Guanabana (Graviola, hoja)
**NYCTAGINACEAE**	*Mirabilis jalapa* L.	Buenas Tardes
**OLACACEAE**	*Heisteria acuminata* (H. & B.) Engler	Chuchuasi (Chuchuhuasi)
**OLACACEAE**	*Ximenia americana* L.	Limoncillo
**OLEACEAE**	*Olea europaea* L.	Hoja de Olivo
**ONAGRACEAE**	*Oenothera rosea* Aiton	Hierba del Dominio, Chupa Sangre
**ORCHIDACEAE**	*Aa paleacea* (H.B.K.) Rchb. f.	Hierba de la Soledad
**ORCHIDACEAE**	*Lycaste gigantea* Lindl.	Caña Caña
**ORCHIDACEAE**	*Stelis* sp.	Cucharilla
**ORCHIDACEAE**	*Stelis eublepharis* Rchb. f.	Boton de Oro (Hierba del Oro, Vara de Oro)
**OXALIDACEAE**	*Oxalis peduncularis* H.B.K.	Chulco
**PAPAVERACEAE**	*Argemone mexicana* L.	Cardo Santo
**PASSIFLORACEAE**	*Passiflora caerulea* L.	Pasionara
**PASSIFLORACEAE**	*Passiflora ligularis* Jus.	Cascara/Hoja de Granadilla
**PASSIFLORACEAE**	*Passiflora quardrangularis* L.	Tumbo
**PHYTOLACCACEAE**	*Petiveria alliacea* L.	Mocura (Mucura)
**PHYTOLACCACEAE**	*Phytolacca bogotensis* H.B.K.	Ilambo, Laylambo, Choco Chocho
**PHYTOLACCACEAE**	*Phytolacca rivinoides* Kunth & Bouché	Guaylango

**PHYTOLACCACEAE**	*Phytolacca weberbaueri* H. Walter	Santo Tome (Santo Tone)
**PINACEAE**	*Pinus patula* Schldl. & Cham.	Pino
**PIPERACEAE**	*Peperomia fraseri* C. DC.	Hierba de la Plata / del Dolar
**PIPERACEAE**	*Peperomia galioides* H.B.K.	Congonilla
**PIPERACEAE**	*Peperomia inaequalifolia* R. & P.	Congona
**PIPERACEAE**	*Piper aduncum* L.	Matico
**PIPERACEAE**	*Piper* cf. *aequale* Vahl.	Mogoquero
**PLANTAGINACEAE**	*Plantago major* L.	Llantén
**PLANTAGINACEAE**	*Plantago sericea* R. & P. subsp. *sericans* (Pilger) Rahn	Paja Blanca
**PLANTAGINACEAE**	*Plantago sericea* R. & P. var. l*anuginosa* Grieseb.	Pajilla Blanca
**POACEAE**	*Arundo donax* L.	Carizo
**POACEAE**	*Cymbopogon citratus* (DC.) Stapf.	Hierba Luisa
**POACEAE**	*Cynodon dactylon* (L.) Persoon	Grama Dulce
**POACEAE**	*Hordeum vulgare* L.	Cebada
**POACEAE**	*Olyra latifolia* L.	Cuña - Cuña
**POACEAE**	*Triticum sativum* L.	Trigo
**POACEAE**	*Zea mays* L.	Chingo
**POLEMONIACEAE**	*Cantua quercifolia* Jus.	Dormidero
**POLYGALACEAE**	*Polygala paniculata* L.	Canchalagua Grande
**POLYGONACEAE**	*Polygonum hydropiperoides* Michaux	Pica Pica (Hierba de Pica Flor)
**POLYPODIACEAE**	*Cheilanthes myriophylla* Desv.	Hierba del Dominio
**POLYPODIACEAE**	*Notholaena nivea* (Poir.) Desv.	Doradilla
**POLYPODIACEAE**	*Polypodium crassifolium* L.	Calaguala (Lengua de Ciervo)
**PUNICACEAE**	*Punica granatum* L.	Cascura de Granada
**RANUNCULACEAE**	*Laccopetalum giganteum* (Wedd.) Ulbrich	Huamanripa (Pacra)
**ROSACEAE**	*Cydonia oblonga* Miller	Membrillo
**ROSACEAE**	*Geum peruvianum* Focke	Valeriana
**ROSACEAE**	*Polylepis racemosa* R. & P.	Quinual
**ROSACEAE**	*Rosa centifolia* L.	Flor de Rosa de Castilla (Rosas)
**ROSACEAE**	*Rubus robustus* C. Presl.	Mora (Zarzamora)
**ROSACEAE**	*Sanguisorba minor* Scop.	Pimpinela
**RUBIACEAE**	*Cinchona officinalis* L.	Cortezas, Cascarilla
**RUBIACEAE**	*Coffea arabica* L.	Cafeto
**RUBIACEAE**	*Uncaria guianensis* (Aubl.) Gmelin	Paraguay
**RUBIACEAE**	*Uncaria tomentosa* (Willdenow ex Roemer & Schultes) DC.	Uña de Gato
**RUIACEAE**	*Morinda citrifolia* L.	Noni
**RUTACEAE**	*Citrus aurantium* L.	Hoja de Naranja
**RUTACEAE**	*Citrus medica* L.	Cidra
**RUTACEAE**	*Gardenia augusta* (L.) Merr.	Margarita
**RUTACEAE**	*Ruta graveolens* L.	Ruda
**SALICACEAE**	*Salix chilensis* Molina	Sauce
**SAPINDACEAE**	*Dodonea viscosa* Jacquin	Chamana
**SAPINDACEAE**	*Sapindus saponaria* L.	Checo
**SAPOTACEAE**	*Pouteria lucuma* (R. & P.) Kuntze	Lucumo
**SAXIFRAGACEAE**	*Escallonia pendula* (R. & P.) Pers.	Chachacon
**SCROPHULARIACEAE**	*Caprania peruviana* Bentham	Arenilla (Flor Arenilla, Te de Indio)
**SCROPHULARIACEAE**	*Escobedia grandiflora* (L.f.) Kuntze	Azafran, Suna
**SCROPHULARIACEAE**	*Galvesia fruticosa* J. Gmelin	Curil, Macacha
**SIMAROUBACEAE**	*Quassia amara* L.	Cuasia
**SMILACACEAE**	*Smilax kunthii* Killip & Morton	Palo de la China
**SMILACACEAE**	*Smilax medica* M. Martens & Galeotti	Zarzaparilla

**SOLANACEAE**	*Brugmansia arborea* (L.) Lagerheim	Floripondio (Micha Churandero, Micha Blanca, Micha Rastrera, Misha)
**SOLANACEAE**	*Brugmansia candida* Persoon	Misha Blanca (Micha Rosada, Rastrera, Blanca)
**SOLANACEAE**	*Brugmansia sanguinea* (R. & P.) D. Don.	Micha Negra (Roja, Rastrera, Blanca)
**SOLANACEAE**	*Cestrum auriculatum* L'Herit	Agrasejo (Hierba Santa, Sangre Maria, Santa Maria)
**SOLANACEAE**	*Datura ferox* L.	Chamico
**SOLANACEAE**	*Jaltomata* sp.	Sémulo
**SOLANACEAE**	*Juanulloa ochracea* Cuatrecasas	Cuya Cuya (semilla)
**SOLANACEAE**	*Lycopersicon peruvianum* (L.) Mill.	Tomate de Monte
**SOLANACEAE**	*Nicotiana rustica* L.	Tabaco negro
**SOLANACEAE**	*Nicotiana tabacum* L.	Hoja de Tabaco (y Tabaco Rubio)
**SOLANACEAE**	*Solanum americanum* Mill.	Baja del Espanto (Hierba Mora)
**SOLANACEAE**	*Solanum mammosum* L.	Torosimuri
**STERCULIACEAE**	*Melochia lupulina Sw.*	Chantilla
**STERCULIACEAE**	*Theobroma cacao* L.	Cascara Cacao
**THELYPTERIDACEAE**	*Thelypteris* cf. *scalaris* (Christ.) Alton	Helecha Macho
**THYMELEACEAE**	*Daphnopsis weberbaueri* Domke	Cholitos (Baron), Hembra
**TROPAEOLACEAE**	*Tropaeolum minus* L.	Mastuerzo
**TYPHACEAE**	*Typha angustifolia* L.	Chace
**ULMACEAE**	*Celtis schippii* Standl.	Palo Huaco
**URTICACEAE**	*Pilea microphylla* (L.) Lieberman	Contra Hierba
**URTICACEAE**	*Urtica magellanica* A. Jussieu ex Poiret	Hortiga (Comun, Ortiga, Ortiga Negra, Ortiga Chica)
**URTICACEAE**	*Urtica* spp.	Hortiga de Leon
**VALERIANACEAE**	*Phyllactis rigida* (R. & P.) Persoon	Estrella
**VALERIANACEAE**	*Valeriana plantaginea* Kunth	Hornamo Morada
**VERBENACEAE**	*Aloysia triphylla* (L. Her.) Britt.	Cedron
**VERBENACEAE**	*Clerodendron* sp.	Brochamelia
**VERBENACEAE**	*Lippia alba (L.) N.E. Br.*	Mestruante
**VERBENACEAE**	*Verbena littoralis* H.B.K.	Verbena
**VIOLACEAE**	*Viola tricolor* L.	Hierba de Pensamiento,Violeta
**XYRIDACEAE**	*Xyris subulata* R. & P.	Hierba del Caballero
**ZINGIBERACEAE**		Chima Pampana
**Various species**		Barbasco
**Commecial mixture**		Siete condores
**Commecial mixture**		Siete hornamos
**Commecial mixture**		Siete mishas
**Commecial mixture**		Siete Raices
**Commecial mixture**		Siete trinzias
**Unidentified**		Chichiricoma
**Unidentified**		Abedul
**Unidentified**		Adan y Eva
**Unidentified**		Alguárate
**Unidentified**		Alquitecta
**Unidentified**		Añisero
**Unidentified**		Cachorillo
**Unidentified**		Candelilla
**Unidentified**		Casiburon
**Unidentified**		Cerrilla
**Unidentified**		Cestisa
**Unidentified**		Chantipa
**Unidentified**		Chapuro
**Unidentified**		Chiricana
**Unidentified**		Doradilla Amarilla
**Unidentified**		Doradilla Blanca

**Unidentified**	Doradilla Negra
**Unidentified**	Estrellado
**Unidentified**	Guancabomba
**Unidentified**	Hierba Chicla
**Unidentified**	Hierba de Carillo
**Unidentified**	Hierba de Conocimiento
**Unidentified**	Hierba de Floricimiento
**Unidentified**	Hierba de Laguna
**Unidentified**	Hierba de Shingo
**Unidentified**	Hierba de Venado
**Unidentified**	Hierba del Amor
**Unidentified**	Hierbas Fuertes
**Unidentified**	Hoja Milagrosa
**Unidentified**	Huarate, Acarate
**Unidentified**	Juaraquice
**Unidentified**	Llamera
**Unidentified**	Madudes
**Unidentified**	Maiz Micha
**Unidentified**	Mandragora
**Unidentified**	Mezclados
**Unidentified**	Michiyo
**Unidentified**	Neumoscada
**Unidentified**	Orel
**Unidentified**	Paígosa, Paigrosa
**Unidentified**	Palo Hueso
**Unidentified**	Paraja
**Unidentified**	Parasaumar, Saumerio
**Unidentified**	Pata Fina
**Unidentified**	Poleo de Pasmo
**Unidentified**	Pusunga
**Unidentified**	Queraños
**Unidentified**	Rio Barbo
**Unidentified**	Seroyil
**Unidentified**	Shivahuaco
**Unidentified**	Sola Juato
**Unidentified**	Tepa Magallon
**Unidentified**	Yorba, Yorbo
**Unidentified**	Zarzachina
