# Supplementary material for: Health for sale: the medicinal plant markets in Trujillo and Chiclayo, Northern Peru
Source: J Ethnobiol Ethnomed. 2007 Dec 10;3:37. doi: 10.1186/1746-4269-3-37 (PMC2245918; doi:10.1186/1746-4269-3-37)
Supplement: Additional File 2 — Quantities of medicinal plants traded in Trujillo and Chiclayo markts (species with more than 1% total sales volume BOLD, exotic species RED) [file 1746-4269-3-37-S2.pdf]

Additional file 2. Quantities of medicinal plants traded in Trujillo and Chiclayo markets

Family Name	Species Name	Common name	Region	Location	Total quantity per week	Sales per week	Total quantity per year	Sales per year
ACANTHACEAE	*Fittonia* spp.	Motalio	Sierra		7	7	364	364
ADIANTACEAE	*Adiantum concinnum* Wild. ex H.B.K.	Culantrillo	Sierra	Huaraz	122	122	6344	6344
ADIANTACEAE	*Pellaea ternifolia C. Chr.*	Cuti Cuti	Sierra		156	156	8112	8112
AIZOACEAE	*Tetragonia crystallina* L'Herit	Hierba de la Señorita	Sierra		34	34	1768	1768
ALGAE	*Giartina* spp.	Algae Marinas			17	17	884	884
ALSTROEMERIACEAE	*Bomarea dulcis* (Hook.) Beauv.	Hierba de Gallo	Sierra		7	7	364	364
AMARANTHACEAE	*Alternanthera brasiliana* (L.) Kuntze	Hierba del Oso	Sierra		17	17	884	884
AMARANTHACEAE	*Alternanthera halmifolia* (Lam.) Standley & Pittier	Morada			17	17	884	884
AMARANTHACEAE	*Alternanthera porrigens* (Jacquin) Kuntze	Moradilla			274	274	14248	14248
AMARANTHACEAE	*Iresine herbstii* Lindley	Zangurache	Sierra		31	31	1612	1612
AMARYLLIDACEAE	*Eustephia coccinea* Cav.	Puma Para	Sierra		63	63	3276	3276
ANACARDIACEAE	***Mauria heterophylla* H.B.K.**	**Feregreco**	**Costa, Sierra**	**Otusco**	**401**	**401**	**20852**	**20852**
ANACARDIACEAE	*Schinus molle* L.	Molle	Costa	Trujillo	43	43	2236	2236
ANNONACEAE	*Annona muricata* L.	Guanabana			217	217	11284	11284
APIACEAE	*Ammi visnaga* (L.) Lam.	Bisnaga	Sierra		17	17	884	884
APIACEAE	*Apium graveolens* L.	Apio	Sierra		89	89	4626	4626
APIACEAE	*Arracacia xanthorrhiza* Bancroft	Racacha	Sierra		7	7	364	364
APIACEAE	*Coriandrum sativum* L.	Culantro			12	12	624	624
APIACEAE	*Daucus montanus* H. & B. ex Spreng.	Hierba de Zorrillo	Sierra		7	7	364	364
APIACEAE	*Foeniculum vulgare* P. Miller	Hinojo	Sierra		89	89	4626	4626
APIACEAE	*Petroselinum crispum* (Miller) A.W. Hill	Perejil	Sierra		58	58	3016	3016
APIACEAE	***Pimpinella anisum* L.**	**Anis**	**Sierra**	**Otusco**	**973**	**973**	**50596**	**50596**
APOCYNACEAE	*Mandevilla* cf. *trianae* Woodson	Bejuco	Sierra		17	17	884	884
APOCYNACEAE	*Nerium oleander* L.	Laurel	Sierra		86	86	4472	4472

**APOCYNACEAE**	*Plumeria rubra* L.	Patchouli	Sierra		17	17	884	884
**APOCYNACEAE**	*Thevetia peruviana* (Pers.) Schum.	Maichil	Costa	Moche	212	212	11024	11024
**APOCYNACEAE**	*Vallesia glabra* (Cav.) Link.	Cuncuno	Sierra		7	7	364	364
**AQUIFOLIACEAE**	*Ilex guayusa* Loes	Citrodora	Sierra		215	215	11180	11180
**ARALIACEAE**	*Oreopanax eriocephalus* Harms	Mano de León	Sierra		57	57	2964	2964
**ARALIACEAE**	*Panax ginseng* Mey.	Ginseng	Sierra		17	17	884	884
**ARECACEAE**	*Bactris* spp.	Chonta	Sierra		7	7	364	364
**ASCLEPIADACEAE**	*Sarcostemma clausum* (Jacquin) Schultes	Marrajudio			10	10	520	520
**ASPHODELACEAE**	***Aloe vera* (L.) Burm f.**	**Hojas de Sabila**		**Chiclayo**	**382**	**382**	**19864**	**19864**
**ASTERACEAE**	*Acanthoxanthium spinosum* (L.) Furreau	Juan Alonso	Costa, Sierra	Moche, Otuzco	70	70	3640	3640
**ASTERACEAE**	*Achillea millefolium* L.	Milenrama	Sierra		41	41	2132	2132
**ASTERACEAE**	*Ambrosia peruviana* Willd.	Altamisa	Costa, Sierra		169	169	8788	8788
**ASTERACEAE**	*Baccharis genistelloides* (Lam.) Pers.	Karqueja	Sierra	Otusco	318	318	16536	16536
**ASTERACEAE**	*Baccharis vaccinioides* H.B.K.	Sigueme Sigueme	Sierra		27	27	1404	1404
**ASTERACEAE**	*Bidens pilosa* L.	Amor Seco			77	77	4004	4004
**ASTERACEAE**	*Calendula officinalis* L.	Calendula	Sierra		7	7	364	364
**ASTERACEAE**	*Chuquiraga spinosa* sp. *huamanpinta* C. Ezcurra	Huamanpinta	Sierra	Huaraz	164	164	8528	8528
**ASTERACEAE**	*Chuquiragua weberbaueri* Tovar	Amaro	Sierra	Otusco	82	82	4264	4264
**ASTERACEAE**	*Cronquistianthus lavandulifolius* DC.	Pulmonaria	Sierra	Otusco	240	240	12480	12480
**ASTERACEAE**	*Cynara cardunculus* L.	Alcachofa	Costa	Moche	232	232	12064	12064
**ASTERACEAE**	*Diplostephium sagasteguii* Cuatrecasas	Hierba del Tigre	Sierra		7	7	364	364
**ASTERACEAE**	*Eupatorium gayanum* Wedd.	Asma chilca	Sierra	Cajamarca	125	125	6500	6500
**ASTERACEAE**	*Eupatorium triplinerve* Wedd.	Chilco	Costa		22	22	1144	1144
**ASTERACEAE**	*Flaveria bidentis* (L.) Kuntze	Mata Gusano	Costa	Trujillo	26	26	1352	1352
**ASTERACEAE**	*Gnaphalium americanum* Mill.	Lechugilla	Sierra	Huaraz	53	53	2756	2756

**ASTERACEAE**	*Loricaria ferruginea* (R. & P.) Wedd.	Palmita	Sierra	Jalca, Huancabamba	10	10	520	520
**ASTERACEAE**	*Loricaria pauciflora* Cuatr.	Palma Bendita	Sierra		27	27	1404	1404
**ASTERACEAE**	*Loricaria thyrsoidea* (Cuatr.) Dillon	Trensilla	Sierra		20	20	1040	1040
**ASTERACEAE**	***Matricaria recutita* L.**	**Manzanilla**	**Sierra**	**Cajamarca y Chiclayo**	**3787**	**3787**	**196924**	**196924**
**ASTERACEAE**	*Monactis flaverioides* H.B.K.	Hierba del Susto	Sierra		92	92	4784	4784
**ASTERACEAE**	***Oritrophium peruvianum* (Lam.) Cuatrec.**	**Vira Vira**	**Costa, Sierra**	**Otusco**	**441**	**441**	**22932**	**22932**
**ASTERACEAE**	*Paranephelius uniflorus* Poepp. & Endl.	Carapa de Chancho	Sierra		41	41	2132	2132
**ASTERACEAE**	***Perezia multiflora* (H. & B.) Lesing**	**Escorcionera**	**Sierra**	**Cajamarca**	**593**	**593**	**30836**	**30836**
**ASTERACEAE**	*Perezia pungens* (H.B.K.) Cas.	Lengua Vaca	Sierra		7	7	364	364
**ASTERACEAE**	*Picrosia longifolia* D. Don	Achicoria	Costa	Moche	120	120	6240	6240
**ASTERACEAE**	*Porophyllum ruderale* (Jacq.) Cas.	Gallinazo	Costa		155	155	8060	8060
**ASTERACEAE**	*Pseudogynoxis cordifolia* (Cass.) Cabr.	San Juan	Sierra		34	34	1768	1768
**ASTERACEAE**	***Schkuhria pinnata* (Lam.) Kuntze**	**Canchalagua**	**Sierra**	**Otusco**	**758**	**758**	**39416**	**39416**
**ASTERACEAE**	*Senecio canescens* (H.B.K.) Cuatrecasas	Oreja de Conejo	Sierra	Sierra	7	7	364	364
**ASTERACEAE**	*Senecio chinogeton* Wedd.	Hornamo Leon Amarillo	Sierra		20	20	1040	1040
**ASTERACEAE**	*Senecio pseudotites* Grieseb.	Arnica	Sierra		21	21	1092	1092
**ASTERACEAE**	*Smallanthus sonchifolius* (Poepp. & Endl) H. Rob.	Yacon	Sierra	Otusco	50	50	2600	2600
**ASTERACEAE**	*Sonchus oleraceus* L.	Serraja	Costa	Costeña	126	126	6552	6552
**ASTERACEAE**	*Spilanthes leiocarpa* DC.	Turre			10	10	520	520
**ASTERACEAE**	*Stevia* sp.	Estevia	Sierra	San Ignacio, Cajamarca	121	121	6292	6292
**ASTERACEAE**	*Tagetes erecta* L.	Claveles Chino	Sierra	Viru	87	87	4524	4524
**ASTERACEAE**	*Tagetes filifolia* Lag.	Anis Serrano	Sierra		24	24	1248	1248
**ASTERACEAE**	*Tanacaetum parthenium* (L.) Sch. Bip.	Santa Marta	Sierra		7	7	364	364

ASTERACEAE	*Taraxacum officinale* Wiggers	Diente de Leon	Costa, Sierra		181	181	9412	9412
ASTERACEAE	*Tegetes elliptica* Sm.	Supequewa			19	19	988	988
ASTERACEAE	*Tesaria integrifolia R. & P.*	Pajaro Bobo	Costa	Moche	104	104	5408	5408
ASTERACEAE	*Trixis cacalioides* H.B.K.	Añasquero Chico	Sierra		7	7	364	364
ASTERACEAE	*Weddelia latifolia* DC.	Chulgan	Sierra		12	12	624	624
BALANOPHORACEAE	*Heliosis cayennensis* (Swartz) Sprengel	Huanarpo	Sierra		36	36	1872	1872
BALSAMIACEAE	*Impatiens balsamina* L.	Balsamina	Sierra		7	7	364	364
BERBERIDACEAE	*Berberis buceronis* J.F. Macbride	Palo Amarillo	Sierra		14	14	728	728
BETULACEAE	*Alnus acuminata* H.B.K.	Aliso	Sierra		21	21	1092	1092
BIGNONIACEAE	*Crescentia cujete* L.	Tutuma	Sierra		39	39	2028	2028
BIGNONIACEAE	*Jacaranda acutifolia* H. & B.	Yarabisca	Sierra		29	29	1508	1508
BIGNONIACEAE	*Tabebuia* spp.	Huayacán	Sierra		7	7	364	364
BIGNONIACEAE	*Tynnanthus scabra* (Hoffm. ex Roem. & Schult.) Schum.	Clavo Huasca	Sierra		24	24	1248	1248
**BIXACEAE**	***Bixa orellana* L.**	**Achote**	**Selva**	**Baja**	**528**	**528**	**27456**	**27456**
**BORAGINACEAE**	***Borrago officinalis* L.**	**Borraja**	**Sierra**	**Otusco**	**360**	**360**	**18720**	**18720**
BORAGINACEAE	*Cordia alliodora* (R. & P.) Oken	Hojas de Ajo Sacha	Selva		33	33	1716	1716
**BORAGINACEAE**	***Cordia lutea* Lam.**	**Flor de Overo**	**Costa**	**Chiclayo**	**960**	**960**	**49920**	**49920**
BORAGINACEAE	*Heliotropium curassavicum* L.	Alacran	Sierra	Cajamarca	14	14	728	728
BORAGINACEAE	*Symphytum* spp.	Confrey	Sierra		7	7	364	364
**BORAGINACEAE**	***Tiquilia paronychoides* (Phil.) Rich.**	**Flor de Arena**	**Costa**		**528**	**528**	**27456**	**27456**
BRASSICACEAE	*Capsella bursa-pastoris* (L.) Medic.	Bolsa de Pastor	Sierra		51	51	2652	2652
BRASSICACEAE	*Cheiranthus cheiri* L.	Alalali	Sierra		7	7	364	364
BRASSICACEAE	*Lepidium meyenii* Walpers	Maca	Sierra		59	59	3068	3068
BRASSICACEAE	*Rorippa nasturtium-aquaticum* (L.) Hayek	Berruco	Sierra		98	98	5096	5096
BROMELIACEAE	*Puya hamata* L.B. Sm.	Hierba de Carnero	Sierra		75	75	3900	3900
**BROMELIACEAE**	***Tillandsia cacticola* L.B. Sm.**	**Siempre Viva**	**Sierra**	**Cajamarca**	**893**	**893**	**46436**	**46436**

**BURSERACEAE**	*Bursera graveolens* (H.B.K.) Triana & Planchon	Palo Santo	Sierra		76	76	3952	3952
**CACTACEAE**	*Echinopsis pachanoi* (Britton & Rose) Friedrich & G. Rowley	San Pedro	Sierra	Huamachuc o	89	89	4628	4628
**CACTACEAE**	*Opuntia ficus-indica* (L.) Miller	Tuna			30	30	1560	1560
**CAMPANULACEAE**	*Siphocampylus angustiflorus* Schlechtendal	Contoya	Sierra		52	52	2704	2704
**CAPPARIDACEAE**	*Capparis crotonoides* H.B.K.	Bichayo	Selva		24	24	1248	1248
**CAPRIFOLIAEAE**	*Lonicera japonica* Thunberg	Madre Selva	Sierra		3	33	1716	1716
**CAPRIFOLIAEAE**	*Sambucus peruviana* HBK	Sauco	Costa, Sierra	Moche	228	228	11856	11856
**CARICACEAE**	*Jacartia digitata* (Poepp. & Endl.) Solms-Lang.	Contra Hechizo	Sierra		47	47	2444	2444
**CARYOPHYLLACEAE**	*Dianthus caryophyllus* L.	Claveles	Costa, Sierra		210	210	10920	10920
**CHENOPODIACEAE**	*Chenopodium ambrosioides* L.	Paico	Costa	Moche	82	82	4264	4264
**CHLORANTHACEAE**	*Hedyosmum racemosum* (R. & P.) G. Don.	Asarcito	Sierra		31	31	1612	1612
**CLETHRACEAE**	*Clethra castaneifolia* Meissner	Hierba del Olvido	Sierra		81	81	4212	4212
**CLUSIACEAE**	*Clusia minor* L.	Churguis	Sierra	Otusco, Uzquil	48	48	2496	2496
**CLUSIACEAE**	*Hypericum laricifolium* Jus.	Hierba de la Fortuna	Sierra		14	14	728	728
**CLUSIACEAE**	*Hypericum silenioides* Jus.	Sentaura	Sierra	Otusco	24	24	1248	1248
**COMMELINACEAE**	*Tripogandra multiflora* (Sw.) Raf.	Cachurros	Sierra		7	7	364	364
**CRASSULACEAE**	*Echeveria peruviana* Meyen	Pinpin	Sierra		78	78	4056	4056
**CUCURBITACEAE**	*Sicana odorifera* (Vell.) Naud.	Secana	Sierra		53	53	2756	2756
**CUPRESSACEAE**	*Cupressus lusitanica* Miller	Cipres	Costa	Moche	40	40	2080	2080
**CYPERACEAE**	*Cyperus articulatus* L.	Varita de San Jose	Costa		24	24	1248	1248
**CYPERACEAE**	*Oreobolos goeppingeri Sues*	Carpintero	Sierra		7	7	364	364
**DIOSCOREACEAE**	*Dioscorea tambillensis*	Papa Semitona			36	36	1872	1872

	Kunth							
**DIOSCOREACEAE**	*Dioscorea trifida* L.f.	Papa Pacta	Sierra	Otusco	69	69	3588	3588
**DIPSACACEAE**	*Scabiosa atropurpurea* L.	Ambarina Negra	Sierra		47	47	2444	2444
**EPHEDRACEAE**	*Ephedra americana* H. & B.	Suelda con Suelda	Sierra	Otusco	305	305	15860	15860
**EQUISETACEAE**	***Equisetum giganteum* (Wedd.) Ulbrich**	**Cola de Caballo**		**Cajamarca**	**3232**	**3232**	**168064**	**168064**
**ERICACEAE**	***Bejaria aestuans* L.**	**Cadillo**	**Costa, Sierra**	**Feriñafen, Chiclayo**	**360**	**360**	**18720**	**18720**
**ERICACEAE**	*Gaultheria erecta* Vent.	Mullaca	Sierra	Otuzco	327	327	17004	17004
**ERYTHROXYLACEAE**	*Erythroxylon coca* Lam.	Coca	Sierra, Montaña	Huamachuc o	277	277	14404	14404
**EUPHORBIACEAE**	*Alchornea castanaeifolia* (Willd.) Jussieu	Ipurura	Costa	Chiclayo	10	10	520	520
**EUPHORBIACEAE**	***Croton lechleri* Muell. Arg.**	**Sangre de Grado**	**Selva**		**5331**	**5331**	**27716l**	**277160**
**EUPHORBIACEAE**	*Hura crepitans* L.	Coco de Abilla			10	10	520	520
**EUPHORBIACEAE**	*Jatropa curcas* L.	Piñones		Norte	47	47	2444	2444
**EUPHORBIACEAE**	*Manhiot esculenta* Crantz	Yuca	Sierra		7	7	364	364
**EUPHORBIACEAE**	***Phyllanthus niruri L., Phyllanthus stipulatus (Raf.) Webster, & Phyllanthus urinaria L.***	**Chanca Piedra**	**Montaña**	**Iquitos, Juanjuil**	**1053**	**1053**	**54556**	**54756**
**EUPHORBIACEAE**	*Ricinus communis* L.	Piñon	Sierra		17	17	884	884
**FABACEAE**	*Acacia senegal* (L.) Willd.	Palo de Goma	Sierra		27	27	1404	1404
**FABACEAE**	*Caesalpinia paipai* R. & P.	Pai Pai	Costa	Moracon	10	10	520	520
**FABACEAE**	*Caesalpinia spinosa* (Molina) Kuntze	Talla	Sierra		115	115	5980	5980
**FABACEAE**	*Cajanus cajan* (L.) Millsp.	Chivato	Sierra		12	12	624	624
**FABACEAE**	*Cassia fistula L.*	Caña Fistula	Sierra	Jaen	27	27	1404	1404
**FABACEAE**	*Copaifera paupera* (Herz.) Dwyer.	Copaiba			10	10	520	520
**FABACEAE**	***Desmodium molliculum* (H.B.K.) DC.**	**Pie de Perro**	**Sierra**	**Otusco**	**813**	**813**	**42276**	**42276**
**FABACEAE**	***Erythrina* sp.**	**Huayruro**	**Montaña**	**Jaén**	**6660 seeds (3 seeds/serving)**	**2220**	**346320**	**115440**
**FABACEAE**	*Lathyrus odoratus* L.	Tacon	Sierra		7	7	364	364
**FABACEAE**	*Leucaena leucocephala* (Lam.) De Wit	Arabisca	Sierra		51	51	2652	2652

**FABACEAE**	*Lupinus mutabilis* Sweet	Flor de Chocho	Sierra		50	50	2600	2600
**FABACEAE**	*Mimosa albida* H. & B.	Tapa Tapa	Costa		19	19	988	988
**FABACEAE**	*Mimosa polydactyla* H. & B.	Sensitiva	Sierra		7	7	364	364
**FABACEAE**	*Mucuna rostrata* Benth.	Habilla	Sierra	Baja	168	168	8736	8736
**FABACEAE**	*Myroxylon balsamum* (L.) Harms.	Quina Quina	Montaña		164	164	8528	8528
**FABACEAE**	*Quassia amara* L.	Cuasia			10	10	520	520
**FABACEAE**	***Senna monilifera* H.S. Irwin & Bowley**	**Sen**	**Costa, Sierra**	**Norteño**	**393**	**393**	**20436**	**20436**
**FABACEAE**	*Senna occidentalis* (L.) Link.	Retana	Sierra		67	67	3484	3484
**FABACEAE**	***Spartium junceum* L.**	**Retama**	**Sierra**	**Otusco**	**853**	**853**	**20436**	**44356**
**FABACEAE**	*Trifolium repens* L.	Trebol	Sierra		48	48	2496	2496
**FABACEAE**	*Zornia reticulata* Sm.	Hierba de la Vibora	Sierra	Cajamarca	44	44	2288	2288
**GENTIANACEAE**	***Gentianella bicolor* (Wedd.) J. Pringle**	**Corpus Way**	**Sierra**	**Otusco**	**592**	**592**	**30784**	**30784**
**GENTIANACEAE**	*Gentianella crasicaulis* J. Pringle	Hojas de Violeta	Sierra	Otuzco	10	10	520	520
**GENTIANACEAE**	*Gentianella dianthoides* (H.B.K.) Fabris	Amargon	Sierra		64	64	3328	3328
**GENTIANACEAE**	***Gentianella graminea* (H.B.K.) Fabris**	**Chinchimali**	**Sierra**		**513**	**513**	**26676**	**26676**
**GENTIANACEAE**	***Gentianella alborosea* (Grimes) Pringle**	**Hercampuri**	**Sierra**	**Junin**	**760**	**760**	**39520**	**39520**
**GENTIANACEAE**	*Gentianella* sp.	Anga Macha	Sierra		31	31	1612	1612
**GERANIACEAE**	*Erodium cicutarium* (L.) L'Herit.	Agujilla			60	60	3120	3120
**GERANIACEAE**	***Geranium ayavacense* Willd ex H.B.K.**	**Pachuchuaca**	**Sierra**	**Otusco**	**620**	**620**	**32240**	**32240**
**GERANIACEAE**	*Pelargonium odoratissimum* (L.) L'Herit.	Malva de olor	Sierra	Cajamarca	49	49	2548	2548
**HIPPOCRATEACEAE**	*Tontelea crassifolia* (Mart.) Spreng.	Bejuco de Montaña	Sierra		7	7	364	364
**JUGLANDACEAE**	*Juglans neotropica* Diels	Nogal	Sierra	Otusco, Huamachuc o	133	133	6916	6916
**LAMIACEAE**	*Hyptis sidifolia* (L´Her.) Briq.	Albaca de Campo	Sierra		162	162	8424	8424
**LAMIACEAE**	*Lavandula angustifolia*	Alhucema	Sierra		41	41	2132	2132

	Miller							
**LAMIACEAE**	*Lepechinia meyenii* (Walpers) Epling	Salvia Real	Sierra		137	137	7124	7124
**LAMIACEAE**	*Marrubium vulgare* L.	Cordon de Muerto	Sierra	Otusco, Uzquil	238	238	12376	12376
**LAMIACEAE**	***Melisa officinalis* L.**	**Toronjil**	**Sierra**	**Cajamarca**	**880**	**880**	**45760**	**45760**
**LAMIACEAE**	***Mentha spicata* L.**	**Menta**	**Sierra**	**Otusco**	**1337**	**1337**	**69524**	**69524**
**LAMIACEAE**	*Mentha x piperita* L.	Poleo	Sierra		57	57	2964	2964
**LAMIACEAE**	*Minthostachys mollis* Griesebach	Muña	Serranilla	Cajamarca	293	293	15236	15236
**LAMIACEAE**	*Ocimum basilicum* L.	Albaca	Costa	Moche	208	208	10816	10816
**LAMIACEAE**	*Origanum majorana* L.	Mejorana	Sierra	Cajamarca	97	97	5044	5044
**LAMIACEAE**	*Origanum vulgare* L.	Oregano de Rama	Sierra	Otuzco	207	207	10764	10764
**LAMIACEAE**	***Otholobium glandulosum* (L.) Grimes**	**Culen**	**Sierra (alta)**	**La Jalca**	**411**	**411**	**21372**	**21372**
**LAMIACEAE**	***Rosmarinus officinalis* L.**	**Romero**	**Costa, Sierra**	**Carjuan, Huaraz, Machuco**	**706**	**706**	**36712**	**36712**
**LAMIACEAE**	*Salvia discolor* H.B.K.	Llatama	Sierra		44	44	2288	2288
**LAMIACEAE**	*Salvia rosmarinifolia* Hort. ex G. Don.	Romero Silvestre	Sierra		37	37	1924	1924
**LAMIACEAE**	*Salvia tubiflora* R. & P.	Hierba del (Mal) Aire	Sierra		105	105	5460	5460
**LAMIACEAE**	*Satureja elliptica* (R. & P.) Briq.	Chipita	Sierra	Loreto, Iquitos	24	24	1248	1248
**LAMIACEAE**	*Satureja pulchella* (H.B.K.) Briquet	Panisara	Sierra	Cajamarca	107	107	5564	5564
**LAMIACEAE**	*Stachys arvensis* L.	Hierba Terrestre	Sierra		7	7	364	364
**LAMIACEAE**	*Thymus vulgaris* L.	Tomillo	Sierra	Central	155	155	8060	8060
**LAURACEAE**	*Aniba roseadora* Ducke	Palo Rosa			12	12	624	624
**LAURACEAE**	*Nectandra* sp. 1	Acharachango	Montaña	Loreto, Iquitos	53 seeds (3 seeds per serving)	53	2756	2756
**LAURACEAE**	*Nectandra* sp. 2	Asmala	Montaña		157 seeds (3 seeds per serving)	157	8164	8164

**LAURACEAE**	***Nectandra reticulata* (R. & P.) Mez.**	**Espingo**	**Montaña**	**Bahua`**	**1343 seeds (3 seeds per serving)**	**448**	**69836**	**23296**
**LICHENES**	*Siphula* sp.	Papelillo	Sierra	Juaraz, Guamachuco	58	58	3016	3016
**LILIACEAE**	*Allium sativum* L.	Ajo Macho	Sierra		14	14	728	728
**LILIACEAE**	*Hesperoziphium niveum* (Rav.) Rav.	Hierba de la Justicia	Sierra		24	24	1248	1248
**LINACEAE**	***Linum sativum* L. & *Linum usitatissimum* L.**	**Linaza**	**Sierra**	**Cajamarca**	**853**	**853**	**44356**	**44356**
**LOGANIACEAE**	***Buddleja utilis* Kraenzl.**	**Flor Blanca**	**Sierra**	**Cajamarca**	**1780**	**1780**	**92560**	**92560**
**LYCOPODIACEAE**	*Huperzia crassa* (H. & B. ex Willd.) Rothm.	Condor	Sierra		57	57	2964	2964
**LYCOPODIACEAE**	*Lycopodium jussiaei* Desv. ex Poir	Hierba del Hombre	Sierra		7	7	364	364
**LYCOPODIACEAE**	*Lycopodium thyoides* H. & B. ex Willd.	Trencilla Roja	Sierra		14	14	728	728
**LYTHRACEAE**	*Cuphea racemosa* (L.f.) Spreng.	Hierba del Coche	Sierra		7	7	364	364
**LYTHRACEAE**	*Cuphea strigulosa* H.B.K.	Hierba del Toro	Sierra	Otusco	314	314	16328	16328
**MALESHERBIACEAE**	*Malesherbia ardens* J.F. Macbr.	Veronica	Sierra	Puno	293	293	15236	15236
**MALPIGHIACEAE**	*Banisteriopsis caapii* (Spruce ex Grieseb.) Morton	Ayahuasca	Selva		84	84	4368	4368
**MALVACEAE**	*Abelmoschus moschatus* Medikus	Hierba de Culebra	Sierra		7	7	364	364
**MALVACEAE**	*Alcea rosea* (L.) Cavanilles	Malva de Lavado			10	10	520	520
**MALVACEAE**	*Malva parviflora* L.	Malva real	Costa	Moche	24	24	1248	1248
**MALVACEAE**	*Malva sylvestris* L.	Malva Blanca	Sierra, Costa	Otusco, Moche, Trujillo	95	95	4940	4940
**MELASTOMATACEAE**	*Brachyotum tyrianthium* Macbride	Sarcilleja	Sierra		14	14	728	728
**MELIACEAE**	*Trichilia* sp.	Pucho	Montaña	Loreto, Iquitos	200 seeds (4 seeds per serving)	50	10400	2600
**MENISPERMACEAE**	*Abuta grandiflora* (Mart.) Sand.	Abuta	Sierra		21	21	1092	1092

**MONIMIACEAE**	***Peumus boldus* Molina**	**Boldo**	**Sierra**	**Chile**	**2328**	**2328**	**121056**	**121056**
**MONIMIACEAE**	*Siparuna muricata* (R. & P.) A. DC.	Añascero	Sierra		82	82	4264	4264
**MORACEAE**	*Brosmium rubescens* Taubert	Palo Sangre	Sierra		72	72	3744	3744
**MORACEAE**	*Ficus carica* L.	Hoja de Higo	Costa	Trujillo	267	267	13884	13884
**MORACEAE**	*Morus alba* L.	Morera	Costa	Moche	55	55	2860	2860
**MUSACEAE**	*Musa x paradisiaca* L.	Platano			21	21	1092	1092
**MYRICACEAE**	*Myricaria dubia* (H.B.K.) McVaugh.	Camu Camu	Sierra		7	7	364	364
**MYRISTICACEAE**	*Myristica fragrans* L.	Nuez moscada	Montaña		100	100	5200	5200
**MYRTACEAE**	***Eucalyptus globulus* Labill.**	**Eucalipto**		**Cajamarca**	**2626**	**2626**	**136552**	**136552**
**MYRTACEAE**	*Eugenia obtusifolia* Cambes.	Lanchi	Sierra		185	185	9620	9620
**MYRTACEAE**	*Myrcianthes discolor* (H.B.K.) Vaughn, *Myrcianthes fragrans* (Sw.) McVaugh	Lanche			33	33	1716	1716
**MYRTACEAE**	*Psidium guajava* L.	Guanabana	Costa	Chiclayo	233	233	12116	12116
**NYCTAGINACEAE**	*Mirabilis jalapa* L.	Buenas Tardes			12	12	624	624
**OLACACEAE**	*Heisteria acuminata* (H. & B.) Engler	Chuchuasi	Sierra		86	86	4472	4472
**OLACACEAE**	*Ximenia americana* L.	Limoncillo	Sierra	Otuzco	29	29	1508	1508
**OLEACEAE**	*Olea europaea* L.	Hoja de Olivo	Sierra		31	31	1612	1612
**ONAGRACEAE**	*Oenothera rosea* Aiton	Chupa Sangre	Sierra		17	17	884	884
**ORCHIDACEAE**	*Aa paleacea* (H.B.K.) Rchb. f.	Hierba de la Soledad	Sierra		7	7	364	364
**ORCHIDACEAE**	*Lycaste gigantea* Lindl.	Caña Caña	Sierra		24	24	1248	1248
**ORCHIDACEAE**	*Stelis* sp.	Cucharilla	Sierra		19	19	988	988
**ORCHIDACEAE**	*Stelis eublepharis* Rchb. f.	Boton de Oro	Costa	Moche	127	127	6604	6604
**OXALIDACEAE**	*Oxalis peduncularis* H.B.K.	Chulco			10	10	520	520
**PAPAVERACEAE**	*Argemone mexicana* L.	Cardo Santo	Sierra		34	34	1768	1768
**PASSIFLORACEAE**	*Passiflora caerulea* L.	Pasionara	Sierra		14	14	728	728
**PASSIFLORACEAE**	*Passiflora ligularis* Jus.	Cascara de Granadilla	Costa	Northern Chiclayo	67	67	3484	3484
**PASSIFLORACEAE**	*Passiflora quardrangularis* L.	Tumbo	Sierra		13	13	676	676
**PHYTOLACCACEAE**	*Petiveria alliacea* L.	Mucura	Costa	Moche	60	60	3120	3120
**PHYTOLACCACEAE**	*Phytolacca bogotensis* H.B.K.	Ilambo	Sierra	Otuzco	35	35	1820	1820

**PHYTOLACCACEAE**	*Phytolacca rivinoides* Kunth & Bouché	Guaylango	Sierra		7	7	364	364
**PHYTOLACCACEAE**	*Phytolacca weberbaueri* H. Walter	Santo Tome	Sierra		14	14	728	728
**PINACEAE**	*Pinus patula* Schldl. & Cham.	Pino			10	10	520	520
**PIPERACEAE**	*Peperomia fraseri* C. DC.	Hierba de la Plata	Sierra		68	68	3536	3536
**PIPERACEAE**	*Peperomia galioides* H.B.K.	Congonilla	Sierra		7	7	364	364
**PIPERACEAE**	*Peperomia inaequalifolia* R. & P.	Congona	Sierra	Otusco, Uzquil	289	289	15028	15028
**PIPERACEAE**	***Piper aduncum* L.**	**Matico**	**Costa**	**Chiclayo**	**1680**	**1680**	**87360**	**87360**
**PIPERACEAE**	*Piper* cf. *aequale* Vahl.	Mogoquero	Sierra		21	21	1092	1092
**PLANTAGINACEAE**	***Plantago major* L.**	**Llantén**	**Costa**	**Moche**	**840**	**840**	**43680**	**43680**
**PLANTAGINACEAE**	*Plantago sericea* R. & P. subsp. *sericans* (Pilger) Rahn	Paja Blanca	Sierra		24	24	1248	1248
**PLANTAGINACEAE**	*Plantago sericea* R. & P. var. *lanuginosa* Grieseb.	Pajilla Blanca	Sierra	Otuzco	12	12	624	624
**POACEAE**	***Cymbopogon citratus* (DC.) Stapf.**	**Hierba Luisa**	**Costa, Sierra**	**Trujillo**	**816**	**816**	**42432**	**42432**
**POACEAE**	***Cynodon dactylon* (L.) Persoon**	**Grama Dulce**	**Costa**	**Moche**	**404**	**404**	**21008**	**21008**
**POACEAE**	*Hordeum vulgare* L.	Cebada			10	10	520	520
**POACEAE**	*Olyra latifolia* L.	Cuña - Cuña			10	10	520	520
**POACEAE**	*Triticum sativum* L.	Trigo	Sierra		7	7	364	364
**POACEAE**	*Zea mays* L.	Chingo	Sierra		27	27	1404	1404
**POLEMONIACEAE**	*Cantua quercifolia* Jus.	Dormidero	Sierra		7	7	364	364
**POLYGALACEAE**	*Polygala paniculata* L.	Canchalagua Grande			10	10	520	520
**POLYGONACEAE**	*Polygonum hydropiperoides* Michaux	Hierba de Pica Flor	Sierra		21	21	1092	1092
**POLYPODIACEAE**	*Cheilanthes myriophylla* Desv.	Hierba del Dominio	Sierra		7	7	364	364
**POLYPODIACEAE**	*Notholaena nivea* (Poir.) Desv.	Doradilla	Sierra	Cajamarca	74	74	3848	3848
**POLYPODIACEAE**	*Polypodium crasifolium L.*	Lengua de Ciervo	Sierra	Huamachuc o	125	125	6500	6500
**PUNICACEAE**	*Punica granatum* L.	Cascura de Granada	Sierra		7	7	364	364
**RANUNCULACEAE**	*Laccopetalum giganteum*	Pacra	Sierra	Huamachuc	292	292	15184	15184

	(Wedd.) Ulbrich			o				
ROSACEAE	*Cydonia oblonga* Miller	Membrillo	Sierra	Sinsicap (Otuzco)	12	12	624	624
**ROSACEAE**	***Geum peruvianum* Focke**	**Valeriana**	**Sierra**	**Otusco**	**413**	**413**	**21476**	**21476**
ROSACEAE	*Polylepis racemosa* R. & P.	Quinual	Sierra		48	48	2496	2496
ROSACEAE	*Rosa centifolia* L.	Rosa de Castilla	Costa	Moche	73	73	3796	3796
ROSACEAE	*Rubus robustus* C. Presl.	Zarzamora	Sierra	Otusco	171	171	8892	8892
**ROSACEAE**	***Sanguisorba minor* Scop.**	**Pimpinela**	**Sierra**	**Otusco**	**663**	**663**	**34476**	**34476**
RUBIACEAE	*Cinchona* spp.	Cascarilla	Sierra		67	67	3484	3484
RUBIACEAE	*Uncaria guianensis* (Aubl.) Gmelin	Paraguay	Sierra		21	21	1092	1092
**RUBIACEAE**	***Uncaria tomentosa* (Willdenow ex Roemer & Schultes) DC.**	**Uña de Gato**	**Selva**		**4530**	**4530**	**235560**	**235560**
RUBICEAE	*Coffea arabica* L.	Café	Sierra		7	7	364	364
**RUIACEAE**	***Morinda citrifolia* L.**	**Noni**		**France**	**27 bottles**	**1350**	**1404 bottles**	**70200**
RUTACEAE	*Citrus aurantium* L.	Hoja de Naranja	Sierra		35	35	1820	1820
RUTACEAE	*Citrus medica* L.	Cidra	Sierra		7	7	364	364
RUTACEAE	*Gardenia augusta* (L.) Merr.	Margarita	Montaña	Jaén	10	10	520	520
**RUTACEAE**	***Ruta graveolens* L.**	**Ruda**	**Costa, Sierra**	**Santa Rosa**	**2013**	**2013**	**104676**	**104676**
SALICACEAE	*Salix chilensis* Molina	Sauce	Costa	Trujillo	35	35	1820	1820
SAPINDACEAE	*Dodonaea viscosa* Jacq.	Chamana	Sierra		36	36	1872	1872
SAPINDACEAE	*Sapindus saponaria* L.	Checo	Sierra		12	12	624	624
SAPOTACEAE	*Pouteria lucuma* (R. & P.) Kuntze	Lucumo	Costa	Morropon	10	10	520	520
SAXIFRAGACEAE	*Escallonia pendula* (R. & P.) Pers.	Chachacon	Sierra	Otusco, Uzquil	24	24	1248	1248
SAXIFRAGACEAE	*Escobedia grandiflora* (L.f.) Kuntze	Suna	Sierra		7	7	364	364
**SCROPHULARIACEAE**	***Caprania peruviana* Bentham**	**Arenilla**	**Montaña**	**Iquitos**	**386**	**386**	**20072**	**20072**
SCROPHULARIACEAE	*Escobedia grandiflora* (L.f.) Kuntze	Azafran	Costa	Moche	12	12	624	624
SCROPHULARIACEAE	*Galvesia fruticosa* J. Gmelin	Curil	Sierra		7	7	364	364
SMILACACEAE	*Smilax kunthii* Killip & Morton	Palo de la China	Sierra		17	17	884	884

**SMILACACEAE**	*Smilax medica* M.Martens & Galeotti	Zarzaparilla	Sierra		61	61	3172	3172
**SOLANACEAE**	*Brugmansia arborea* (L.) Lagerheim	Micha Rastrera	Sierra		105	105	5460	5460
**SOLANACEAE**	*Brugmansia candida* Persoon	Misha Blanca			43	43	2236	2236
**SOLANACEAE**	*Brugmansia sanguinea* (R. & P.) D. Don.	Misha Roja			27	27	1404	1404
**SOLANACEAE**	***Cestrum auriculatum* L'Herit**	**Agrasejo**	**Costa**	**Norte**	**679**	**679**	**35308**	**35308**
**SOLANACEAE**	*Datura ferox* L.	Chamico	Sierra, Selva		20	20	1040	1040
**SOLANACEAE**	*Jaltomata* sp.	Sémulo	Sierra		93	93	4836	4836
**SOLANACEAE**	*Juanulloa ochracea* Cuatrecasas	Cuya-Cuya	Sierra	Jaen	41	41	2132	2132
**SOLANACEAE**	*Lycopersicon peruvianum* (L.) Mill.	Tomate de Monte	Sierra		7	7	364	364
**SOLANACEAE**	*Nicotiana rustica* L.	Tabaco negro			10	10	520	520
**SOLANACEAE**	*Nicotiana tabacum* L.	Tabaco	Costa, Sierra, Montaña		125	125	6500	6500
**SOLANACEAE**	*Solanum americanum* Mill.	Hierba Mora	Sierra	Uzkil	27	27	1404	1404
**SOLANACEAE**	*Solanum mammosum* L.	Torosimuri	Sierra		7	7	364	364
**STERCULIACEAE**	*Melochia lupulina* Sw.	Chantilla	Sierra		19	19	988	988
**STERCULIACEAE**	*Theobroma cacao* L.	Cascara Cacao	Sierra	Jaén	7	7	364	364
**THELYPTERIDACEAE**	*Thelypteris scalaris* (Christ) Alston	Helecha Macho	Sierra	Ortusco, Uzkil	7	7	364	364
**THYMELEACEAE**	*Daphnopsis weberbaueri* Domke	Cholitos	Sierra	Pucallpa	7	7	364	364
**TILIACEAE**	***Tilia platyphyllos* L.**	**Tilo**		**Chile**	**679**	**679**	**35308**	**35308**
**TROPAEOLACEAE**	*Tropaeolum minus* L.	Mastuerzo	Costa	Todas Costas	19	19	988	988
**TYPHACEAE**	*Typha angustifolia* L.	Chace			10	10	520	520
**ULMACEAE**	*Celtis schippii* Standl.	Palo Huaco	Sierra		38	38	1976	1976
**URTICACEAE**	*Pilea microphylla* (L.) Lieberman	Contra Hierba	Sierra (baja)	Huancayo	47	47	2444	2444
**URTICACEAE**	***Urtica magellanica* A. Jussieu ex Poiret**	**Hortiga Comun**	**Sierra**	**Otuzco**	**526**	**526**	**27352**	**27352**
**URTICACEAE**	*Urtica* spp.	Hortiga de Leon	Sierra	Otuzco	10	10	520	520
**VALERIANACEAE**	*Phyllactis rigida* (R. & P.) Persoon	Hierba de Estrella			33	33	1716	1716

**VALERIANACEAE**	*Valeriana plantaginea* Kunth	Hornamo Morada			20	20	1040	1040
**VERBENACEAE**	***Aloysia triphylla* (L. Her.) Britt.**	**Cedron**	**Sierra**	**Otusco**	**480**	**480**	**24960**	**24960**
**VERBENACEAE**	*Clerodendron* sp.	Brochamelia	Costa	Moche	73	73	3796	3796
**VERBENACEAE**	*Lippia alba* (L.) N.E. Br.	Mestruante			12	12	624	624
**VERBENACEAE**	*Verbena littoralis* H.B.K.	Verbena	Costa, Sierra	Moche	72	72	3744	3744
VIOLACEAE	*Viola tricolor L.*	Hierba del Pensamiento	Sierra	Otusco	97	97	5044	5044
**XYRIDACEAE**	*Xyris subulata* R. & P.	Hierba del Caballero			10	10	520	520
**ZYGOPHYLLACEAE**	*Tribulus terrestris* L.	Carizo	Costa	Moche	30	30	1560	1560
**INDET.**		7 condores	Sierra		7	7	364	364
**INDET.**		7 hornamos	Sierra		7	7	364	364
**INDET.**		7 michas	Sierra		14	14	728	728
**INDET.**		7 trinzias	Sierra		7	7	364	364
**INDET.**		Adan y Eva	Sierra		7	7	364	364
**INDET.**		Ajojero	Sierra		7	7	364	364
**INDET.**		Alquitecta	Sierra		10	10	520	520
**INDET.**		Barbasco		Iquitos	10	10	520	520
**INDET.**		Cachorillo	Sierra		7	7	364	364
**INDET.**		Candelilla	Sierra		7	7	364	364
**INDET.**		Casiburon	Sierra	Otusco	24	24	1248	1248
**INDET.**		Cembraca	Sierra		7	7	364	364
**INDET.**		Cerrilla	Sierra		7	7	364	364
**INDET.**		Cestisa	Sierra		7	7	364	364
**INDET.**		Chapuro			12	12	624	624
**INDET.**		Chichiricoma	Sierra	Loreto, Iquitos	24	24	1248	1248
**INDET.**		Chiricana	Sierra		7	7	364	364
**INDET.**		Chochocon	Sierra		27	27	1404	1404
**INDET.**		Estrellado	Sierra		34	34	1768	1768
**INDET.**		Guancabomba	Sierra		24	24	1248	1248
**INDET.**		Guayaba	Sierra		7	7	364	364
**INDET.**		Hierba Chicla	Sierra		7	7	364	364
**INDET.**		Hierba de Carillo	Sierra		7	7	364	364
**INDET.**		Hierba de Conocimiento	Sierra		7	7	364	364
**INDET.**		Hierba de			10	10	520	520

		Floricimiento						
**INDET.**		Hierba de Laguna	Sierra		7	7	364	364
**INDET.**		Hierba de Shingo			12	12	624	624
**INDET.**		Hierba de Venado	Sierra		12	12	624	624
**INDET.**		Hierba del Amor			10	10	520	520
**INDET.**		Hierbas Fuertes	Sierra		7	7	364	364
**INDET.**		Hoja de Abedul	Sierra	Otusco	48	48	2496	2496
**INDET.**		Hoja Milagrosa	Sierra		7	7	364	364
**INDET.**		Huarate	Sierra	Sinsicap (Otuzco)	55	55	2860	2860
**INDET.**		Juaraquice	Sierra		7	7	364	364
**INDET.**		Llamera			10	10	520	520
**INDET.**		Madudes	Sierra		7	7	364	364
**INDET.**		Maiz Micha	Sierra		7	7	364	364
**INDET.**		Mandragora	Sierra		7	7	364	364
**INDET.**		Mano Poderosa	Sierra		7	7	364	364
**INDET.**		Mezclados			10	10	520	520
**INDET.**		Michiyo	Sierra		7	7	364	364
**INDET.**		Mishquina	Sierra		7	7	364	364
**INDET.**		Neumoscada	Sierra		7	7	364	364
**INDET.**		Orel	Sierra		7	7	364	364
**INDET.**		Paígosa	Sierra		19	19	988	988
**INDET.**		Palo Hueso	Sierra		7	7	364	364
**INDET.**		Paraja			12	12	624	624
**INDET.**		Parasaumar			20	20	1040	1040
**INDET.**		Pata Fina			10	10	520	520
**INDET.**		Poleo de Pasmo	Sierra		7	7	364	364
**INDET.**		Pusunga			10	10	520	520
**INDET.**		Queraños	Sierra		7	7	364	364
**INDET.**		Rio Barbo	Sierra	Iquitos	17	17	884	884
**INDET.**		Ruminaria			10	10	520	520
**INDET.**		Seroyil	Sierra		7	7	364	364
**INDET.**		Shivahuaco	Sierra		7	7	364	364
**INDET.**		Siete Raices			10	10	520	520

**INDET.**		Sola Juato	Sierra		7	7	364	364
**INDET.**		Te Amargo	Sierra		7	7	364	364
**INDET.**		Tepa Magallon	Sierra		24	24	1248	1248
**INDET.**		Yorba	Sierra		14	14	728	728
**INDET.**		Zarzachina	Sierra		7	7	364	364
					Total soles	71552		3721172
						650.55 per vendor per week		33828.83 per vendor per year
					**Total US$**	**22790.13 per week**		**1185086.62 per year**
						**207.18 per vendor per week**		**10773.52 per vendor per year**
						**91160.51 per month**		
						**828.73 per vendor per month**		
